# Urine proteomics for discovery of improved diagnostic markers of Kawasaki disease

**DOI:** 10.1002/emmm.201201494

**Published:** 2012-12-20

**Authors:** Alex Kentsis, Andrew Shulman, Saima Ahmed, Eileen Brennan, Michael C Monuteaux, Young-Ho Lee, Susan Lipsett, Joao A Paulo, Fatma Dedeoglu, Robert Fuhlbrigge, Richard Bachur, Gary Bradwin, Moshe Arditi, Robert P Sundel, Jane W Newburger, Hanno Steen, Susan Kim

**Affiliations:** 1 Division of Hematology/Oncology, Department of Pediatric Oncology, Dana-Farber Cancer Institute, Boston Children's Hospital, Harvard Medical SchoolBoston, MA, USA; 2 Rheumatology Program, Division of Immunology, Boston Children's Hospital, Harvard Medical SchoolBoston, MA, USA; 3 Department of Pathology, Proteomics Center, Boston Children's Hospital, Harvard Medical SchoolBoston, MA, USA; 4 Department of Laboratory Medicine, Boston Children's Hospital, Harvard Medical SchoolBoston, MA, USA; 5 Division of Emergency Medicine, Boston Children's Hospital, Harvard Medical SchoolBoston, MA, USA; 6 Department of Pediatrics, Infectious and Immunological Diseases Research Center, Cedars-Sinai Medical CenterLos Angeles, CA, USA; 7 Department of Cardiology, Boston Children's Hospital, Harvard Medical SchoolBoston, MA, USA

**Keywords:** biomarker, Kawasaki disease, mass spectrometry, vasculitis, urinary proteome

## Abstract

Kawasaki disease (KD) is a systemic vasculitis of unknown etiology. Absence of definitive diagnostic markers limits the accuracy of clinical evaluations of suspected KD with significant increases in morbidity. In turn, incomplete understanding of its molecular pathogenesis hinders the identification of rational targets needed to improve therapy. We used high-accuracy mass spectrometry proteomics to analyse over 2000 unique proteins in clinical urine specimens of patients with KD. We discovered that urine proteomes of patients with KD, but not those with mimicking conditions, were enriched for markers of cellular injury such as filamin and talin, immune regulators such as complement regulator CSMD3, immune pattern recognition receptor muclin, and immune cytokine protease meprin A. Significant elevations of filamin C and meprin A were detected in both the serum and urine in two independent cohorts of patients with KD, comprised of a total of 236 patients. Meprin A and filamin C exhibited superior diagnostic performance as compared to currently used markers of disease in a blinded case-control study of 107 patients with suspected KD, with receiver operating characteristic areas under the curve of 0.98 (95% confidence intervals [CI] of 0.97–1 and 0.95–1, respectively). Notably, meprin A was enriched in the coronary artery lesions of a mouse model of KD. In all, urine proteome profiles revealed novel candidate molecular markers of KD, including filamin C and meprin A that exhibit excellent diagnostic performance. These disease markers may improve the diagnostic accuracy of clinical evaluations of children with suspected KD, lead to the identification of novel therapeutic targets, and allow the development of a biological classification of Kawasaki disease.

## INTRODUCTION

Kawasaki disease (KD) or mucocutaneous lymph node syndrome is an acute, systemic vasculitis of children that presents with prolonged fever and mucocutaneous inflammation, including inflammation of the oral mucosa, non-exudative conjunctivitis, rash, extremity changes and cervical lymphadenopathy that is usually unilateral (Burns et al, 1991). Although Kawasaki disease has an annual incidence of about 1 in 10,000 in American and European populations, it is the most common cause of acquired paediatric heart disease in the developed world and remains a major medical problem because its signs and symptoms mimic many other childhood febrile illnesses (Baker et al, 2009; Taubert et al, 1991). In addition, the prevalence of KD is particularly high in Asia; 1 in 100 Japanese children under the age of 5 years develop Kawasaki disease (Nakamura et al, 2010).

Delays in accurate diagnosis lead to increased mortality and morbidity from complications of Kawasaki disease (Suda et al, 2011; Wilder et al, 2007). In particular, without timely treatment, as many as 25% of patients may develop coronary artery dilatation or aneurysms, with the associated risk of long-term morbidity or death (McCrindle et al, 2007). Importantly, no pathognomonic test exists for the early identification and diagnosis of KD (Dedeoglu & Sundel, 2007; Gedalia, 2007). The use of clinical algorithms has improved the diagnosis of Kawasaki disease, but their accuracy remains limited (Yellen et al, 2010).

Attempts to improve the reliability of clinical evaluations of Kawasaki disease have focused on clinical and general laboratory markers of inflammation (Chow et al, 1993; Ebihara et al, 2005; Lin et al, 1992; Peng et al, 2006; Suganami et al, 2008). However, their performance is inadequate, likely because of their non-specific relationship to the pathophysiology of KD, which is thought to be caused by an interaction between an infectious trigger and an exaggerated inflammatory response (Rowley et al, 2008).

In the current study, we employed a discovery-based approach, seeking to identify these pathophysiological alterations on a proteomic scale. We studied urine because of its abundance and relative analytic simplicity as compared to serum. Previously, we and others have successfully used high accuracy mass spectrometry to measure urine proteomes with sufficient depth to identify local and systemic biomarkers, and to discover improved diagnostic markers of disease (Adachi et al, 2006; Kentsis et al, 2010; Oetting et al, 2006; Pisitkun et al, 2006; Rai et al, 2005; Woroniecki et al, 2006; Zimmerli et al, 2008).

The goal of our study was to discover and validate diagnostic markers of Kawasaki disease in a prospective paediatric cohort. By using high accuracy mass spectrometry proteome profiling of urine specimens collected from children with suspected KD, we analysed the differences in individual urine proteomes. Candidate diagnostic markers were validated in the urine and serum in two independent cohorts of patients with KD using enzyme-linked immunosorbent assays (ELISAs). Their diagnostic performance was then assessed in a blinded, prospective study of children with suspected KD.

## RESULTS

### Mass spectrometry proteomics for discovery of Kawasaki disease markers

During the course of this study, we enrolled 107 subjects who presented with fever and concern for possible KD, based on established diagnostic criteria (Newburger et al, 2004). In agreement with previous studies of the epidemiology and presentation of KD, our study population was predominantly male, with a mean age of 3 years and with the presenting signs and symptoms described in Table 1. Fifty-three patients (49%) were ultimately diagnosed with KD. All patients with KD received treatment with high-dose aspirin and intravenous gammaglobulin, with 16 patients (30%) requiring repeat treatment due to lack of initial clinical response, including one patient (2%) who initially responded to therapy but developed recurrent disease 5.5 months following initial presentation. Thirty-three of 54 patients without Kawasaki disease (61%) were found to have non-specific viral syndrome, with the remaining patients found to have a variety of conditions that may mimic KD (Table 2).

**Table 1:** Presenting signs, symptoms and diagnostic studies of 107 patients with suspected of Kawasaki disease

Characteristic	Final diagnosis	
	
	Non-KD *n* = 54	KD *n* = 53
Gender (% male)^c^	27 (50)	39 (73)
Race (%)		
Caucasian	38 (70.3)	28 (52.8)
African American	4 (7.4)	3 (5.6)
Asian^c^	4 (7.4)	12 (22.6)
Other	8 (14.8)	7 (20.1)
Age (years)^c^	5 [3.3,6.7]	3.3 [1.8,5]
Duration of fever, days	6 [5,7]	6 [5,7]
Number of primary criteria^a^,^b^,^c^	2 [1,3]	4 [4,5]
Conjunctivitis (%)^c^	31 (57)	51 (96)
Mucositis (%)^c^	24 (44)	51 (96)
Rash (%)^c^	31 (57)	51 (96)
Extremity changes (%)^c^	14 (26)	45 (85)
Lymphadenopathy (%)^c^	12 (22)	28 (53)
Pyuria (%)^c^	5 (9.8)	16 (30)
Peripheral WBC (K cells/mm^3^)^c^	8.8 [6.5,12.7]	14.7 [12,17]
Hgb (g/dl)^c^	11.6 [11.1,12.2]	10.7 [10,11.5]
Platelet (K cells/mm^3^)^c^	297 [215,351]	402 [345,478]
Na (mmol/L)	135 [133,137]	134 [132,135.5]
CRP (mg/dl)^c^	4.9 [2.2, 8.3]	9.2 [6.3, 16.7]
ESR (mm/h)^c^	44 [27.8, 69]	86 [61,96.3]
ALT (unit/L)^c^	16 [11.5, 33]	28 [14,104.5]
Albumin (g/dl)^c^	3.8 [3.6, 4]	3.5 [3.3, 3.8]
Maximum coronary artery *Z*-score	n/a	1.4 [0.8, 1.9]
Incomplete presentation^b^ (%)	n/a	11 (20.7)

Values are reported as frequency (percent) or median [IQR], where appropriate.

a Primary criteria: fever ≥5 days, conjunctivitis, oropharyngeal findings, rash, extremity changes, adenopathy.

b Incomplete presentation (see text for description); Hgb, hemoglobin; Na, sodium; CRP, C-reactive protein; ESR, erythrocyte sedimentation rate; ALT, alanine aminotransferase. Pyuria was defined as having >10 white blood cells/high-powered field.

c Difference between KD and non-KD groups statistically significant (*p* < 0.05).

**Table 2:** Final diagnosis of the 107 study patients

Final diagnosis	Number of patients
Kawasaki disease	53
Viral syndrome	33
Adenovirus	6
Serum sickness	3
Pyelonephritis	2
group A streptococcal pharyngitis	2
Cytomegalovirus	1
Epstein-Barr virus	1
Group A streptococcal pharyngitis	1
Lyme disease	1
Otitis media	1
Pneumonia	1
Respiratory syncytial virus	1
Systemic arthritis	1

**Figure 1:**
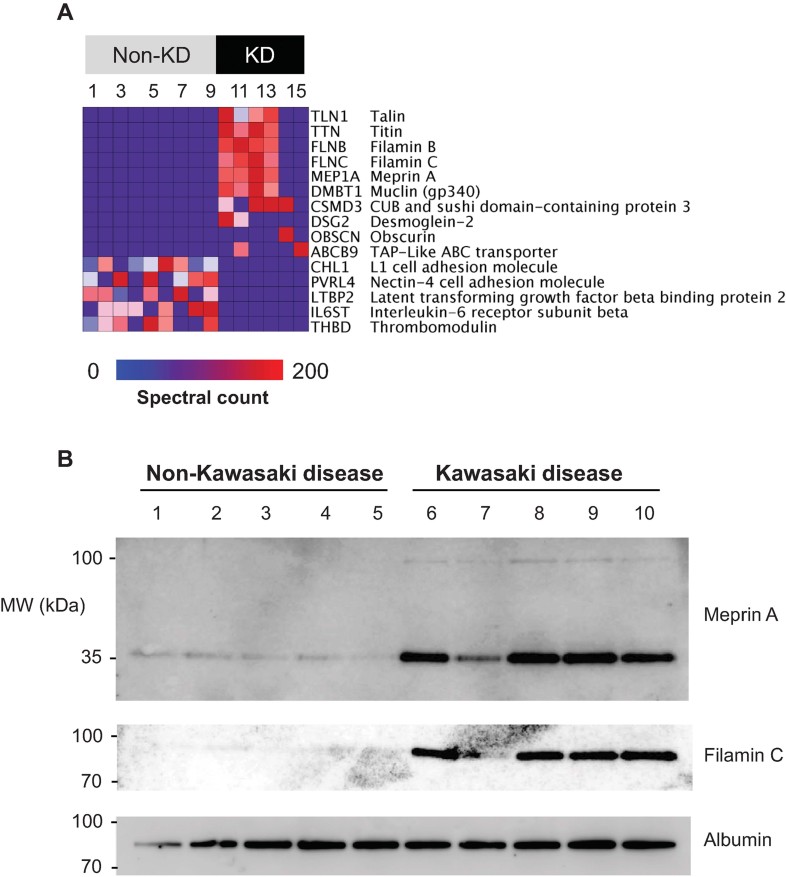
Patients with Kawasaki disease exhibit a unique urine proteome that is distinct from patients without KD or commonly present urinary proteins Heatmap of the 15 individual urinary proteomes (columns) showing the results of Bayesian analysis of the top 10 proteins (rows) that are detected in patients with KD as compared to those without. The top 5 proteins that are detected in patients without KD as compared to those with KD are shown for comparison. Blue-to-red colour gradient represents the number of MS/MS spectra (spectral count) that corresponds to urinary protein abundance.Western immunoblot analysis of meprin A and filamin C in urine, demonstrating enrichment of meprin A and filamin C in the urine of patients with KD as compared to patients without KD. Note that meprin A and filamin C are detected predominantly as partial-length isoforms, with apparent molecular weights of 35 and 80 kDa, respectively. Albumin serves as the loading control.

Candidate diagnostic markers of KD were identified based on the analysis of 15 specimens collected at the onset of the study and chosen based on availability: 6 KD specimens (3 without and 3 with coronary artery dilatation), 6 non-KD specimens (2 with non-specific viral syndromes, 3 with adenovirus, and 1 with pyelonephritis), and 3 matched specimens collected from patients with KD 1 month following complete response to treatment (convalescent KD). This analysis identified 2131 unique proteins, with the tissue and physical origin of the aggregate urine proteomes similar to previous studies (Kentsis et al, 2009). Analysis of the three comparison groups led to the identification of more than 190 proteins in the urine of patients with KD, but not in any of the patients without KD or in those whose KD had resolved completely (Supporting Information Table 1). We analysed the abundance of candidate KD markers to identify those that are most enriched in patients with KD, ranking them in order of relative abundance and prevalence (Fig 1). The identified markers include a variety of proteins associated with endothelial and myocardial cell injury such as filamin and titin, and immune regulators such as DMBT1 and meprin A. Many of the detected markers are high molecular weight proteins, which are probably enriched in the urine of patients with KD as processed and/or truncated proteins, as confirmed for meprin A and filamin C by Western immunoblotting (Fig 1A). The discovered proteomes are provided in Supporting Information Table 2 (processed data), and the raw data, in accordance with the MIAPE standard, and are openly available at Peptide Atlas (http://www.peptideatlas.org).

### Validation of filamin C and meprin A using immunoassays

**Figure 2:**
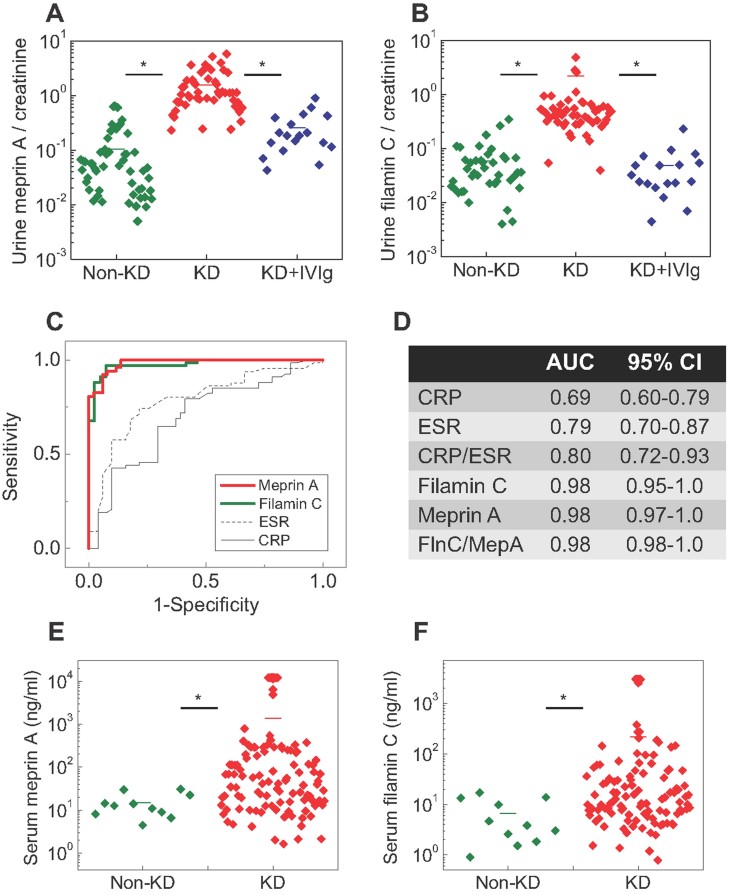
Patients with Kawasaki disease but not those with mimicking conditions have significantly elevated serum and urine levels of meprin A and filamin C, which exhibit superior diagnostic performance in a blinded study of patients suspected of KD **A,B.** otplots of urine concentrations measured using specific ELISAs of meprin A (**A**) and filamin C (**B**) in a blinded case-control study of patients suspected of KD, demonstrating significantly elevated concentrations of meprin A and filamin C in patients with KD (red) as compared to those with non-KD conditions (green) and patients with KD upon receiving IVIg treatment (blue). *p* = 2.0E−8 and 1.3E−17 for filamin C and meprin A, respectively. Horizontal bars represent means for each comparison group. Note the logarithmic scale of meprin A and filamin C concentrations.**C.** Receiver operating characteristics of urine meprin A (red) and filamin C (green), as compared to common markers, ESR (dashed black) and blood CRP (solid black).**D.** Receiver operating characteristic area under the curve (AUC) values and their 95% confidence intervals (CI) for the measured diagnostic markers.**E,F.** Dotplots of serum concentrations measured using specific ELISAs of meprin A (**E**) and filamin C (**F**) in patients with KD (red) as compared to patients with non-KD mimicking conditions (green). *p* = 1.2E−7 and 3.9E−4 for filamin C and meprin A, respectively.

In order to validate selected candidate KD markers, we chose to analyse the levels of filamin C and meprin A because of their biological functions that may play a role in KD *vide infra* and because of availability of commercial ELISAs. We chose to validate candidate markers in urine because of its availability. To assess the KD diagnostic performance of filamin C and meprin A, we prospectively measured their concentrations in the urine of patients, with investigators blinded to the patients' final diagnosis. Patients studied as part of the discovery phase of our study were not included in this validation. We did not find statistically significant differences in total urine protein concentration in the cohort under study (Supporting Information Fig 1). However, urine concentrations of both meprin A and filamin C were significantly elevated in patients with KD as compared to those without, even when corrected for variability in total urine concentration based on urine creatinine (mean filamin C of 19.2 *vs.* 3.7 ng/ml, and mean meprin A of 50.2 *vs.* 5.6 ng/ml, two-tailed *t*-test *p* = 2.0E−8 and 1.3E−17, respectively, Fig 2A and B, Supporting Information Table 3). Controlling for age, sex, race and duration of fever using logistic regression did not affect the statistical significance of the elevations of meprin A and filamin C. Notably, we found that urine meprin A and filamin C were also significantly elevated in patients with incomplete presentations of KD, meeting only three out of four major diagnostic criteria, as compared to those without KD (mean filamin C of 17.0 *vs.* 3.7 ng/ml, and mean meprin A of 41.5 *vs.* 5.6 ng/ml, two-tailed *t*-test *p* = 2.7E−6 and 2.2E−6, respectively).

### Diagnostic performance of validated markers of Kawasaki disease

We analysed the diagnostic performance of meprin A and filamin C for all patients in the validation cohort using receiver operating characteristic (ROC) analysis. The ROC curves for these markers exhibited superior diagnostic performance as compared to the currently used laboratory markers such as the erythrocyte sedimentation rate (ESR) and blood C-reactive protein (CRP), with urine creatinine-normalized meprin A and filamin C having an area under the curve value of 0.98 with 95% confidence intervals of 0.97–1 and 0.95–1, respectively (Fig 2C and D). The diagnostic performance of either urine meprin A or filamin C was superior to that of ESR, CRP or the combination of CRP/ESR (Fig 2D). The combination index of filamin C and meprin A did not lead to statistically significant improvements over the use of individual markers alone (FlnC/MepA; Fig 2D). Notably, urine meprin A and filamin C concentrations did not correlate with the presence of pyuria, CRP or ESR (Supporting Information Figs 1–5).

**Figure 3:**
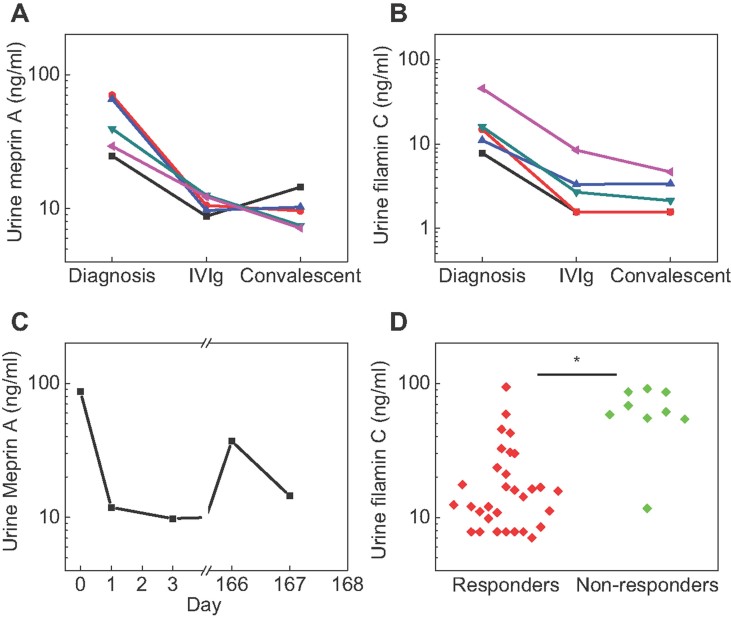
Urine filamin C and meprin A correlate with disease activity in patients with KD **A,B.** Urine meprin A (**A**) and filamin C (**B**) levels in five patients with KD, as measured in matched specimens collected at diagnosis, 24–48 h after treatment, and 1 month after complete clinical response.**C.** Urine meprin A level in one patient who experienced recurrence of KD 5.5 months after initial presentation.**D.** Scatter plot showing urine filamin C levels in patients who responded to initial therapy (red, responders) *versus* those who required repeat treatment (green, non-responders). *p* = 0.0015.

In addition, we assessed the relationship between meprin A and filamin C and response to therapy, by measuring their urine concentrations in matched serial specimens. These were collected at diagnosis prior to initiation of therapy, 24–48 h after treatment with high dose aspirin and intravenous gammaglobulin, and 1 month after complete clinical response to treatment in five patients for whom matched specimens could be collected. In all patients studied, urine meprin A and filamin C levels correlated with response to treatment (one-way ANOVA *p* = 0.043 and 0.031, respectively, Fig 3A and B).

In particular, we were able to measure urine meprin A in one patient with KD who initially responded to treatment, but whose disease recurred 5.5 months after initial presentation. We observed recurrent elevation of urine meprin A associated with the relapse of Kawasaki disease (Fig 3C). Likewise, we found that patients who required repeat treatment with intravenous gammaglobulin due to the lack of initial clinical response had significantly higher levels of filamin C at presentation than patients who responded to initial therapy (mean 64 *vs.* 20 ng/ml, two-tailed *t*-test *p* = 0.0015, Fig 3D).

Encouraged by these findings, we sought to extend the validation of meprin A and filamin C to an independent cohort of patients. Consequently, we obtained archived specimens collected from patients with Kawasaki disease that would be available for immediate study. Because urine specimens were not available, we obtained 112 serum specimens of patients with KD, collected as part of the recent Paediatric Heart Network study, and compared them to patients initially suspected to have KD but ultimately diagnosed with non-KD febrile illnesses (Fig 2E and F). Using ELISAs, we found that both meprin A and filamin C were significantly elevated in the serum of patients with KD as compared to non-KD controls (mean filamin C of 217 *vs.* 6.6 ng/ml, and mean meprin A of 1363 *vs.* 14.8 ng/ml, respectively, two-tailed *t*-test *p* = 1.2E−7 and 3.9E−4, respectively, Supporting Information Table 4).

**Figure 4:**
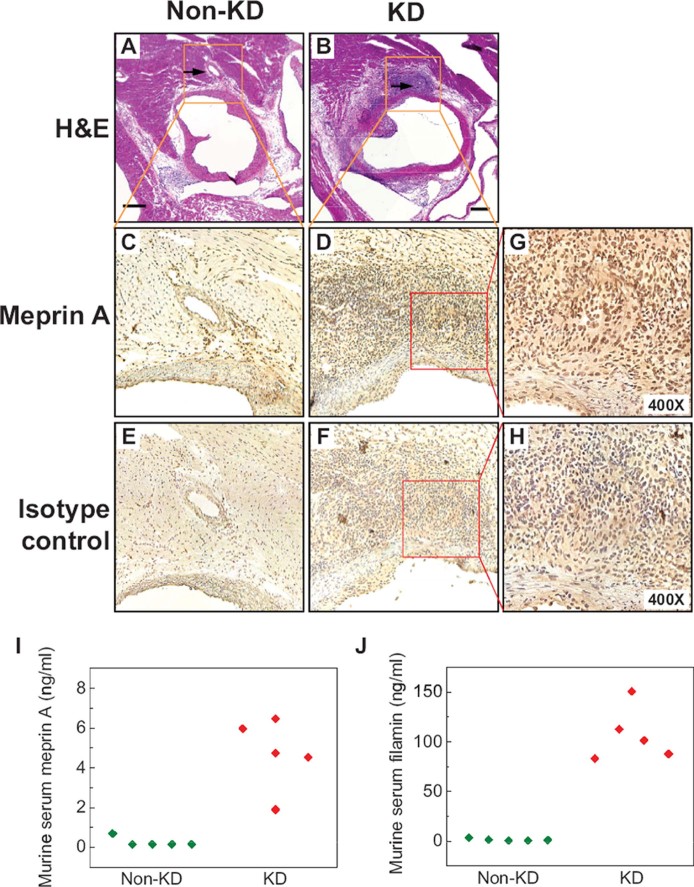
Meprin A is enriched in coronary artery lesions in a mouse model of Kawasaki disease **A,B.** Micrographs of hematoxylin and eosin-stained sections of the aortic root and coronary arteries of control (**A**) and LCWE-injected mice (**B**) demonstrating severe aortitis with intimal proliferation leading to concentric obstruction in the LCWE-injected but not control animals. Arrows point to the normal (**A**) and diseased (**B**) coronary arteries.**C,D.** Micrographs of meprin A immunohistochemistry-stained inset areas of the sections of coronary arteries demonstrating enrichment of meprin A in the mononuclear infiltrates of coronary arteries in LCWE-injected (**D**) but not control (**C**) animals.**E,F.** Micrographs of isotype control-stained sections of coronary arteries in LCWE-injected (**F**) and control (**E**) mice.**G,H.** High-magnification micrographs of the inset areas of meprin A (**G**) and isotype control-strained (**H**) sections of the coronary arteries in LCWE-injected mice.**I,J.** Serum levels of meprin A (**I**) and filamin (**J**) are elevated in the LCWE-injected (red) as compared to control (green) mice. Scale bar is 250 µM.

Since meprin A is a protease that regulates a variety of immune cytokines, we investigated the potential involvement of meprin A in the pathogenesis of Kawasaki disease using a mouse model of coronary arteritis that reproduces several features of KD (Lehman et al, 1988). In particular, moribund mice develop systemic mononuclear vasculitis that leads to myocarditis, aortitis and coronary arteritis with intimal proliferation, characteristic of Kawasaki disease. Using immunohistochemical analysis, we found that meprin A was enriched in the vascular lesions of mice with coronary arteritis but not in control mice using specific meprin A antibodies (Fig 4A–H). Likewise, we found that levels of circulating meprin A and filamin C were significantly elevated in the serum of mice with coronary arteritis as compared to control mice (mean 4.7 *vs.* 0.3 ng/ml, and 107.2 *vs.* 1.4 ng/ml, two-tailed *t*-test *p* = 0.0053 and 0.00098, respectively, Fig 4I and J).

## DISCUSSION

Kawasaki disease, an acute idiopathic vasculitis of children, causes significant morbidity and mortality if not diagnosed and treated expeditiously. The use of clinical algorithms in combination with echocardiography has improved the accuracy of diagnostic evaluations of Kawasaki disease. In conjunction with prompt treatment, this has led to significant reductions in mortality and complications from coronary aneurysms. However, major diagnostic challenges remain because the clinical criteria used to diagnose KD are not specific for this condition, and a significant subset of children with KD lacks several of the cardinal manifestations of the disease.

Several studies have sought to identify biomarkers of this condition with the goal of improving the diagnostic accuracy of evaluations of possible KD. Acute phase reactants such as peripheral blood white cell count, ESR and CRP levels are the most clinically useful. However, these markers remain inadequate in terms of their specificity and sensitivity (Huang et al, 2010; Xiu-Yu et al, 2010), as confirmed in our study (Fig 2C and D). Recent attempts to identify improved diagnostic markers, such as osteoprotegerin, natriuretic peptide and vascular endothelial growth factor produced limited improvements, likely as a result of insufficient specificity for the distinct immune mechanisms that characterize KD (Ebata et al, 2011; Kaneko et al, 2011; Simonini et al, 2005).

By leveraging the high accuracy and sensitivity of recently developed mass spectrometry approaches, we sought to discover more accurate and sensitive diagnostic markers. We analysed the urine proteomes of patients with KD as compared to those initially suspected to have KD but ultimately proved to have other febrile illnesses. This allowed us to construct a molecular pathophysiological profile of Kawasaki disease comprising of over 190 candidate KD markers (Fig 1 and Supporting Information Table 1). These molecules include potential markers of endothelial and myocardial injury (talin, filamin, desmoglein, obscurin and titin), leukocyte activation (AMICA1, CAECAM, CXCL12, GDF15 and LAIR1), pathogen immune recognition (DMBT1, ABCB9) and cytokine regulation (CSMD3, meprin A).

We found several immune regulatory molecules to be uniquely present in the urine of patients with KD. Among these was meprin A, a metalloprotease that functions in the activation and degradation of inflammatory cytokines, which have been implicated in the pathogenesis of KD, including IL-1 and IL-6 (Chow et al, 1993; Herzog et al, 2005). Similarly, DMBT1, also known as muclin or gp340, is an innate immune scavenger receptor that recognizes a variety of bacterial and viral antigens (Madsen et al, 2010). Finally, ABCB9, also known as TAPL, is a transporter that functions in immune antigen presentation (Bangert et al, 2011). Many of the identified KD marker candidates, if properly validated as we have done here for meprin A and filamin C, may represent not only diagnostic markers, but also novel therapeutic targets. Taken together, the identified proteomes, including both raw and processed data, available openly at Peptide Atlas (http://www.peptideatlas.org), and containing 190 novel candidate KD markers listed in Supporting Information Tables 1 and 2, provide a molecular physiological profile of Kawasaki disease. Interactions of the components of the innate and adaptive immune responses in patients implicated by this molecular profile may further elucidate pathogenic mechanisms mediating Kawasaki disease.

Importantly, using a prospective, blinded study of patients with suspected KD, we confirmed that filamin C and meprin A are significantly elevated in the serum and urine of patients with KD but not those with a variety of mimicking conditions (Fig 2). Both markers demonstrated superior diagnostic performance as compared to the currently used laboratory tests (Fig 2). In contrast to filamin A and filamin B, the high expression of filamin C in myocytes (Bonnemann et al, 2003) suggests that filamin C represents a sensitive and specific marker of the subclinical myocarditis that accompanies KD. Indeed, markers of frank cardiomyocyte injury such as troponin have not been found to correlate with clinical or echocardiographic evidence of myocarditis (Checchia et al, 2001; Sato et al, 2011). In addition, elevated levels of filamin C in patients with KD who did not respond to initial therapy as compared to those with complete response suggest that filamin C is a marker of Kawasaki disease activity (Fig 3D). Meprin A and filamin C were also elevated in patients with incomplete presentations of KD, suggesting that these markers may be used to improve the diagnosis of incomplete presentations of KD.

Similarly, meprin A is a protease that regulates a variety of inflammatory cytokines, including biologically active IL-1β, a key pro-inflammatory cytokine (Herzog et al, 2005), polymorphisms of which have been associated with resistance to treatment of KD (Weng et al, 2010). Thus, meprin A may contribute to the initiation, propagation or compensatory immune mechanisms of KD. The potential contributions of meprin A to the pathophysiology of KD are emphasized by its enrichment in the coronary lesions in a mouse model of KD (Fig 4) and correlation with disease severity in patients with KD (Fig 3). Though meprin A is expressed by the kidney and leukocytes (Bond et al, 2005), meprin A elevations in the urine of patients with KD do not appear to relate to the presence of pyuria that can accompany KD (Supporting Information Fig 2). Also, elevations of urine meprin A or filamin C in patients suspected of Kawasaki disease do not appear to correlate to acute phase reactants such as ESR or CRP (Supporting Information Figs 3–6).

The mechanisms by which these discovered KD markers accumulate in the urine of patients and their relationship to the pathophysiology of KD are important directions for future work. We attempted to mitigate the stochastic effects of instrumental sampling in proteomics by using Bayesian statistics, but cannot rule out the possibility that the discovered molecular pathophysiological profile of KD may both omit significant and include irrelevant proteins. In addition, meprin A and filamin C will require further studies in patients with renal or urologic disease or extreme dehydration, and development of their clinical-grade assays will require normalization for potential variations in total urine protein concentration. Ultimately, testing of meprin A and filamin C in multi-institutional, interventional studies of KD will be necessary for their clinical implementation.

In summary, the work presented here opens many potential approaches for improving the diagnosis of Kawasaki disease, elucidating its pathophysiology and directing therapy. In particular, validation of meprin A and filamin C as specific and sensitive markers of Kawasaki disease using commonly available ELISAs enables their clinical use to improve the accuracy and timeliness of diagnosis of KD. In addition, the described molecular physiological profiles and validated diagnostic markers should allow for a biological classification of Kawasaki disease that will lead to improved patient stratification and allow for individualized treatment, as also recently suggested by Cohen and colleagues (Ling et al, 2011). More broadly, the approach presented here advances a proteomic profiling paradigm designed specifically for direct translation to clinical practice, with applications in a wide variety of common and rare human conditions (Kentsis, 2011; Kentsis et al, 2009).

## MATERIALS AND METHODS

### Study participants

The study was conducted over a 39 month period beginning in January 2009 at a tertiary care paediatric hospital and approved by the Boston Children's Hospital Committee on Clinical Investigation. Patients younger than 18 years of age who were being evaluated for possible Kawasaki disease were enrolled according to clinical history and physical examination. Patients were excluded if they had pre-existing neoplastic, renal or urologic disease or were pregnant. The paediatric emergency medicine or rheumatology physicians obtained written consent from caregivers and assent for children older than 7 years of age. Informed consent was obtained from all subjects and the research conformed to the principles set out in the WMA Declaration of Helsinki and the NIH Belmont Report.

### Study design

Our study was conducted in two phases. For the discovery phase, urine samples from six patients with Kawasaki disease, including both patients with and without coronary artery ectasia, were compared with urine samples from six patients initially suspected of Kawasaki disease but with the final diagnosis of febrile illnesses mimicking Kawasaki disease (two with non-specific viral syndromes, three with adenovirus and one with pyelonephritis). We also included in the discovery analysis three intra-individual control specimens collected from patients with Kawasaki disease after completing treatment and resolution of symptoms.

The validation phase was comprised of the analysis of two independent cohorts. The first cohort was based on patients evaluated for possible Kawasaki disease, but before the determination of the final diagnosis. The second cohort utilized serum specimens collected as part of the Pediatric Heart Network Study of Kawasaki disease (Newburger et al, 2007).

For all study patients, urine was collected as clean-catch samples at the time of clinical evaluation. Specimens were cleared by centrifugation according to standard methods, and were labelled with a study number such that all analysis was blinded. Specimens were stored at −80°C within 6 h of collection. Blood specimens for serum collection were clotted and centrifuged according to standard methods, with the serum stored at −80°C within 30 min of collection.

### Outcome measures

Final diagnosis was determined by paediatric rheumatology physicians of a single tertiary care institution according to published diagnostic criteria for Kawasaki disease (Newburger et al, 2004). For patients with at least 4 days of fever, the diagnosis of Kawasaki disease was established using either four or more principal clinical criteria for Kawasaki disease or a coronary artery *z*-score of 2.5 or more for the proximal right coronary artery or the left anterior descending coronary artery, as measured by two-dimensional echocardiography (Newburger et al, 2004). For patients with incomplete presentations of KD meeting <4 diagnostic clinical criteria, the diagnosis of KD was established according to the American Heart Association Guidelines (Newburger et al, 2004). For patients enrolled as part of the Pediatric Heart Network Study, the final diagnosis was determined using identical criteria (Newburger et al, 2007). For patients initially suspected of KD but ultimately diagnosed with non-KD febrile illnesses, the diagnosis of KD was excluded by the lack of four primary diagnostic criteria of KD. For patients with <4 primary diagnostic criteria, the diagnosis of KD was excluded by the absence of elevations of CRP or ESR, or by the absence of supplemental laboratory criteria, as published (Newburger et al, 2004). For patients who were found to not have Kawasaki disease, a diagnosis of non-specific viral syndrome was assigned based on clinical evaluation, if no specific pathogen was identified. For patients who were not hospitalized, the outcome was confirmed by telephone 6–8 weeks after evaluation using scripted questions or from medical chart review to ascertain whether the patient had any subsequent medical care. All studied patients received a final outcome. Clinical and laboratory data was tracked using standardized case report forms.

### Urine proteome analysis

For the discovery of candidate markers of Kawasaki disease, thawed 5 ml urine aliquots were fractionated using ultracentrifugation, protein precipitation, SDS–PAGE, and reverse-phase liquid chromatography, as described in detail (Kentsis et al, 2009). Individual urine protein fractions were subjected to liquid chromatography tandem mass spectrometry using a nanoflow HPLC system (Eksigent, Dublin, CA) coupled to the hybrid linear ion trap-Fourier transform ion cyclotron resonance mass spectrometer (LTQ FT Ultra, Thermo Scientific, Waltham, MA). For each MS/MS spectrum, the 200 most intense peaks were extracted and searched against the human International Protein Index database (version 3.69, http://www.ebi.ac.uk/IPI) by using MASCOT (version 2.1.04, Matrix Science). Assessment of identification accuracy was carried out by searching a decoy database composed of reversed protein sequences of the target IPI database (Elias & Gygi, 2007). Only proteins identified on the basis of two or more unique peptides were included in the analysis, at a false discovery rate of <1% at the peptide level. Analysed individual urine proteomes are openly available at Peptide Atlas (http://www.peptideatlas.org).

### Laboratory measurements and filamin C and meprin A immunoassays

Blood chemistry, immunoassay tests such as CRP and urine creatinine measurements were performed using the COBAS and Modular P systems (Roche Diagnostics). ESRs were measured using the Excyte 40 automated analyser (Vital Diagnostics). Urinalysis measurements were performed using the Clinitek Atlas automated analyser (Siemens). Concentrations of filamin C and meprin A were measured using enzyme-linked immunosorbent assays (USCN Life Science, Wuhan, China). The lowest detection limits of the assays were 0.1 and 0.06 ng/ml, respectively, with the coefficient of variation for the quality control specimens of <10% for both assays. Specificity was confirmed using purified synthetic proteins, and apparent immunoassay interference effects in urine were corrected by serial dilution. For Western immunoassays, 1 ml urine aliquots were precipitated by adding trichloroacetic acid to 10% w/v and dithiothreitol to 10 mM final concentration, and incubating on a rotating shaker at 4°C overnight. Precipitated proteins were sedimented by centrifugation at 16,000 *g* for 20 min at 4°C, washed twice with cold acetone, air-dried, and dissolved in 0.1 ml of RIPA buffer. Protein concentrations were determined by using bicinchoninic acid-copper reduction (Bio-Rad) and 30 µg protein aliquots were denatured in Laemmli buffer at 95°C for 5 min, resolved using SDS–PAGE, transferred to PVDF membrane (Pierce), blocked and probed by using primary antibodies against meprin A (clone 364312, R&D Systems) or filamin C (clone A01, Abnova). Bound antibodies were detected by using horseradish peroxidase-conjugated secondary antibodies, SuperSignal West Dura reagent (Pierce), and the ImageQuant LAS 4000 chemiluminescence imaging system (GE Healthcare), according to manufacturer's instructions.

**The paper explained**

PROBLEM:

In spite of years of intensive study, accurate diagnosis and understanding of the causes of Kawasaki disease remain elusive. As a consequence, delayed or inaccurate diagnosis causes significant complications and long-term injury and hinders the development of improved therapies.

RESULTS:

Using recently developed techniques for analysing thousands of protein molecules in the urine of patients with Kawasaki disease, and those with conditions that routinely mimic Kawasaki disease, we identified signatures of distinct biological processes that are associated with Kawasaki disease. Using tests readily amenable for routine medical use, we found that two discovered markers of Kawasaki disease, meprin A and filamin C, can be used to identify patients with Kawasaki disease with excellent accuracy.

IMPACT:

Development of clinical tests using these markers may improve the diagnostic accuracy of evaluations of children with suspected Kawasaki disease and lead to the development of improved treatments.

### Analysis of murine model of coronary arteritis and meprin A immunohistochemistry

We used an established murine model of coronary arteritis based on intraperitoneal injection of the cell wall extract of group B *Lactobacillus casei* (LCWE) (Lehman et al, 1988). Group B *L. casei* were grown and its cell wall extract was prepared as described (Schulte et al, 2009). Briefly, 6-week old C57/BL6 mice were injected with 250 µg of LCWE in phosphate buffered saline (PBS) or with saline alone. Fourteen days later, mice were sacrificed, and coronary arteries were identified in serial sections (7 µm) fixed with formalin and stained with hematoxylin and eosin. For the immunohistochemical analysis, sections were pre-treated with 0.3% hydrogen peroxide in PBS for 30 min. Meprin A (clone F-20, Santa Cruz Biotechnology, Santa Cruz, CA) or isotype control antibody (goat serum, Santa Cruz Biotechnology) was applied in 0.5% bovine serum albumin in PBS at 1:100 for 1 h. Slides were then washed and biotinylated anti-goat horseradish peroxidase conjugated secondary antibody (Vector Lab, Burlingame, CA) was applied at 1:500 for 30 min, washed and stained with streptavidin conjugated horseradish peroxidase (BD Biosciences, San Diego, CA) at 1:1000 for 30 min. Immunohistochemical staining was detected using the SK-4100 DAB kit, as per manufacturer's instructions (Vector Lab).

### Statistical analysis

Discovery urine proteomes were assembled by parsimonious protein grouping, as described (Kentsis et al, 2009), with the individual peptide counts summed to calculate protein spectral counts. We used Bayesian statistics, as implemented in QSpec (Choi et al, 2008), to analyse protein spectral counts to identify proteins that are statistically significantly enriched at the 1% false discovery threshold in samples from patients with Kawasaki disease, but not in samples from non-Kawasaki disease patients or intra-personal control samples of patients with Kawasaki disease after completion of treatment. Observed peptide spectral counts were normalized to the lengths of the corresponding expected proteins. No assumptions about sample independence were made, allowing for inclusion of paired specimens, which significantly improve discovery yields (Kentsis et al, 2010). ROCs and multivariate linear regression models were calculated using standard methods (STATA, version 10.1, StataCorp). All statistical tests were two-tailed using comparisons of log-transformed measurements.

## Supplementary material

As a service to our authors and readers, this journal provides supporting information supplied by the authors. Such materials are peer reviewed and may be re-organized for online delivery, but are not copy-edited or typeset. Technical support issues arising from supporting information (other than missing files) should be addressed to the authors.
