# Supplementary material for: Urine proteomics for discovery of improved diagnostic markers of Kawasaki disease
Source: EMBO Mol Med. 2012 Dec 20;5(2):210–20. doi: 10.1002/emmm.201201494 (PMC3569638; doi:10.1002/emmm.201201494)
Supplement: Supplementary file 1 [file emmm0005-0210-SD1.pdf]

EMBO
Molecular Medicine

Urine proteomics for discovery of improved diagnostic markers of Kawasaki disease

Andrew Shulman, Saima Ahmed, Eileen Brennan, Michael C. Monuteaux, Young-Ho Lee, Susan Lipsett, Joao A. Paulo, Fatma Dedeoglu, Robert Fuhlbrigge, Richard Bachur, Gary Bradwin, Moshe Arditi, Robert P. Sundel, Jane W. Newburger, Hanno Steen, Susan Kim and Alex Kentsis

Corresponding author: Alex Kentsis, Dana-Farber Cancer Institute, Children's Hospital Boston, Harvard Medical School

---

Review timeline:

Submission date:	22 April 2012
Editorial Decision:	21 May 2012
Revision received:	20 September 2012
Editorial Decision:	17 October 2012
Revision received:	26 October 2012
Accepted:	06 November 2012

Transaction Report:

(Note: With the exception of the correction of typographical or spelling errors that could be a source of ambiguity, letters and reports are not edited. The original formatting of letters and referee reports may not be reflected in this compilation.)

1st Editorial Decision 21 May 2012

Thank you for the submission of your manuscript to EMBO Molecular Medicine. We have now heard back from the three referees whom we asked to evaluate your manuscript. Although the referees find the study to be of potential interest, they also raise a number of concerns that should be convincingly addressed in a major revision of the present manuscript.

As you will see from the reports below, although the referees find the study to be of potential interest, they nevertheless recommend some additional data and explanations in order to substantiate your claims on both clinical and biological levels. Given these evaluations, I would like to give you the opportunity to revise your manuscript, with the understanding that the referees' concerns must be fully addressed and that acceptance of the manuscript would entail a second round of review.

I look forward to seeing a revised form of your manuscript as soon as possible.

Yours sincerely,

Editor
EMBO Molecular Medicine

***** Reviewer's comments *****

Referee #1 (Comments on Novelty/Model System):

My major concern about this manuscript is that the authors did not normalize the protein data to urinaty creatinine, and since urinary volumes depend on hydration status, changes in protein content

© EMBO

may be due to that effect. The fact that all protein biomarkers were elevated in KD, concerns me that the children with KD may have been more dehydrated

Referee #1 (Other Remarks):

This is an interesting study which reports the urine proteomics discovery of biomarkers for KD diagnosis. As it stands the manuscript although interesting, is not very clear and is in need of some improvements and careful attention to substantiate their claims of both the clinical utility and biological significance of the findings.

a. Figure 1 shows the heatmap of the top 15 urinary proteins' spectral counts. The author analyzed normalized protein spectral counts but no details of the normalization were described. Were the spectral counts normalized to the molecular weight or the amino acid length of the found urinary proteins?

b. The biomarkers (Fig.1) were all of high molecular weights, e.g. TLN1 (2541 AA), TTN (34350 AA), FLNC (2725 AA), MEP1A (774 AA), gp340 (2413 AA), CSMD3 (3707 AA), DSG2 (1118 AA). Most likely these proteins cannot "survive" as full length proteins in the urine. In addition, all of these proteins have multiple isoforms. Western blot analysis is suggested to identify which of the fragments are true biomarkers.

c. From Fig 1, all of the protein candidate biomarkers were up in KD. Is there any biomarker candidates were down in KD? There is the possibility that urinary concentrations were higher in patients with KD. It would be important to include each sample's spectral count data for the 190 candidate proteins as supplementary data sets.

d. The logic why only filamin C and meprin A were chosen for validation is not very clear.

e. Urine protein analyses suffer from major variances where biological issues include dilution of urine by different hydration states of the urine donors. Therefore, it is essential that the ELISA quantification of protein candidates in urine needs to be normalized to creatinine.

f. There are little data linking potential biomarkers as novel therapeutic targets, despite the title of the article.

g. The claim that the biomarkers (talin, filamin, desmoglein, obscurin, titin) are endothelial and cardiac musce specific is not necessarily borne out by where these proteins are found, and may be related to the type of isoform present.

Referee #2 (Other Remarks):

Children with Kawasaki disease (KD) can develop coronary artery aneurysms, especially when standard therapy fails to halt inflammation. Interest in identifying patients at greatest risk of non-response and prognosis at early disease onset is high, with the aim of targeting them for more aggressive initial treatment. Kim and colleagues have explored the possibility of identifying sensitive peptide markers from the urine samples of suspected KD patient so early diagnosis can be affirmed and effective treatment can be designed. Although this study is of great interest to better understanding mechanisms underlying KD pathogenesis and clinic treatment, the current study is very preliminary and substantial works should be performed to validate their claims. In short, I am supportive for its publication, pending the following issues being addressed satisfactorily:

Specific points:
1. The number of KD cohort (63 patients) is too small to make any conclusions. In order to make a valid claim, it is required to analyze at least 200 patients;

2. The authors should also specific the protocol for urine sample collection as peptide spectra of urine change according to circadian;
3. To validate the fidelity of the markers, it is recommended to analyze the urine peptide spectra of suspected KD patients before treatment such as corticosteroids and after the treatment to validate the correlation. Since the authors claim for identification of therapeutic targets of KD using their current protocol, the comparison of urine peptide spectra before and after treatment is essential;
4. The authors should elaborate why elevated levels of filamin C and meprin in urine sample could be used as diagnostic markers for KD.

Referee #3 (Other Remarks):

The authors report the results from a urine proteomics study to identify candidate markers of Kawasaki disease. From analysis of the mass spectrometry spectral count data, the authors demonstrate that among the 2000 proteins identified, two proteins of interest tracked with KD. The two proteins, Meprin A and Filamin C, were tested in additional patient sets with commercially available ELSIA kits. The protein levels were clearly elevated in the KD patient serum. In addition, Meprin A was elevated in serum obtained from a mouse model of KD. Overall the paper provide for an expanded set of molecular components that may be associated with the onset of KD. The MS experiments are excellent, and the data are reported to MIAPE standards and raw MS data are provided. Overall this is a very strong paper, and I have only minor comments for the authors to consider.

The authors report the re-appearance of elevated levels of meprin A in the one patient that relapsed. What about levels of filamin C? It appears that the levels did not elevate (or were not tested for?). What, if any, implications do the authors think this has for the predictive role of filamin c?

What were the filamin C levels in the murine serum? The filamin C levels were elevated in human serum. Could this be related to the murine model, or a lowered predictive power of filamin c concentration in plasma (see above comment)? This may be worth some discussion?

Would the authors expect altered levels of IL-1 and IL-6 with an elevated level of meprin A in KD patents? Could this be tested for in the murine model?

1st Revision - authors' response 20 September 2012

Referee #1
Comment a. *Figure 1 shows the heatmap of the top 15 urinary proteins' spectral counts. The author analyzed normalized protein spectral counts but no details of the normalization were described. Were the spectral counts normalized to the molecular weight or the amino acid length of the found urinary proteins?*

> Response: As the referee points out, the apparent spectral counts for each protein are related both to the protein abundance and length; the latter acting to increase the number of observed peptides for longer proteins. For this reason, we used an analysis procedure based on the QSpec algorithm, which includes normalization of the observed peptide spectral counts to the corresponding lengths of the expected intact proteins (Significance analysis of spectral count data in label-free shotgun proteomics. Choi H, Fermin D, Nesvizhskii AI. Mol Cell Proteomics. 2008 Dec;7(12):2373-85). We have revised the manuscript to clarify this point (revised text in blue, pg. 18).

Comment b. *The biomarkers (Fig.1) were all of high molecular weights, e.g. TLN1 (2541 AA), TTN (34350 AA), FLNC (2725 AA), MEP1A (774 AA), gp340 (2413 AA), CSMD3 (3707 AA), DSG2 (1118 AA). Most likely these proteins cannot "survive" as full length proteins in the urine. In addition, all of these proteins have multiple isoforms. Western blot analysis is suggested to identify which of the fragments are true biomarkers.*

Response: We agree with the referee that specific forms of these proteins are likely present in the patients with Kawasaki disease, either due to the expression of specific isoforms or their proteolytic processing. We have now examined the urine specimens of 5 subjects with Kawasaki disease, as compared to 5 patients with other causes of fever for intact proteins by Western immunoblotting. We focused on the two validated KD markers, meprin A and filamin C, with the expected molecular weights of full-length isoforms of 84 and 291 kDa, respectively. Consistent with the referee's prediction, we found that meprin A exists predominantly as a 35 kDa species, with a minor population of the full-length 84 kDa meprin A, and filamin C is detected as an 80 kDa species. The mechanism of the generation of these shorter protein forms, as for example by splicing or proteolysis that occurs specifically in patients with KD, is an important direction of future work. We have now revised Fig. 1 to include these results (Fig. 1B; revised text in blue, pg. 6).

Comment c. *From Fig 1, all of the protein candidate biomarkers were up in KD. Is there any biomarker candidates were down in KD? There is the possibility that urinary concentrations were higher in patients with KD. It would be important to include each sample's spectral count data for the 190 candidate proteins as supplementary data sets.*

Response: We have also found proteins enriched specifically in the patients without KD as compared to those with KD, such as peptides derived from thrombomodulin and interleukin-6 receptor. We have now revised Fig. 1 to include these proteins (revised Fig. 1A). We do not believe that enrichment of KD-specific markers is due to increased urine concentration in patients with KD, as demonstrated by Western immunoblotting (revised Fig. 1B), measurements of urine creatinine and total protein (Supp. Fig. 1). As requested by the referee, we have also provided a supplementary dataset that includes the mass spectral counts of the detected peptides (Supplemental File 1).

Comment d. *The logic why only filamin C and meprin A were chosen for validation is not very clear.*

Response: We chose to validate meprin A because it is a metalloprotease that functions in the activation and degradation of inflammatory cytokines which have been implicated in the pathogenesis of KD, including IL-1 and IL-6 (Chow et al, 1993; Herzog et al, 2005). Filamin C is appealing as a specific marker of KD because of its high expression in the heart, and myocarditis is frequently involved in KD. We have revised the manuscript to clarify these explanations (text in blue, pg. 11).

Comment e. *Urine protein analyses suffer from major variances where biological issues include dilution of urine by different hydration states of the urine donors. Therefore, it is essential that the ELISA quantification of protein candidates in urine needs to be normalized to creatinine.*

Response: We thank the referee for this important suggestion, and have now measured creatinine concentrations in the urine specimens in our study (revised Supp. Fig. 1). Likewise, we have normalized all urine meprin A and filamin C concentrations (ng/ml) to their corresponding urine creatinine concentrations (mg/ml), and present the normalized values in the revised Figs. 1A and B. In spite of differences in urine creatinine concentration between patients with and without KD (about 2-fold; revised Supp. Fig. 1), patients with KD have significantly higher creatinine-normalized meprin A and filamin C values (more than 10-fold, revised Figs. 1A and B).

Comment f. *There are little data linking potential biomarkers as novel therapeutic targets, despite the title of the article.*

Response: We agree with the referee and have revised the title accordingly.

Comment g. *The claim that the biomarkers (talin, filamin, desmoglein, obscurin, titin) are endothelial and cardiac musce specific is not necessarily borne out by where these proteins are found, and may be related to the type of isoform present.*

Response: We agree with the referee and have clarified this description in the revised manuscript (revised text in blue, pg. 10).

Referee #2

Comment 1. *The number of KD cohort (63 patients) is too small to make any conclusions. In order to make a valid claim, it is required to analyze at least 200 patients;*

Response: We agree with the referee about the importance of studying large numbers of patients suspected of Kawasaki disease, both to faithfully capture intrinsic heterogeneity of this syndrome, as well as the variability of the conditions that can mimic Kawasaki disease in clinical practice. During the past 6 months, we have been able to enroll 44 additional subjects into our study, and now include them in the analysis presented in the revised manuscript, which includes a total of 236 subjects. The inclusion of the additional 44 patients actually improved the diagnostic performance of meprin A and filamin C. Given that the 95% confidence intervals of the diagnostic performance (ROC AUC) of meprin A and filamin C are 0.97-1 and 0.95-1, respectively (revised Fig. 2D), we believe that inclusion of additional patients will not lead to significant changes beyond these already excellent values. While we are continuing the enrollment of additional patients, given the rare incidence of this disease (even at our referral institution), enrollment of 200 patients will take more than 18 months. We hope that the referee will agree with us that the current performance and this length of time is beyond the scope of the requirements for the current manuscript, and that the excellent diagnostic performance observed in the expanded cohort will be sufficient for publication of our revised manuscript. Importantly, the patient cohort included in the revised manuscript includes both the intrinsic heterogeneity of Kawasaki disease (typical vs. atypical presentation in Table 1), as well as the variable mimicking conditions (Table 2), suggesting that meprin A and filamin C are indeed specific markers of Kawasaki disease.

Comment 2. *The authors should also specific the protocol for urine sample collection as peptide spectra of urine change according to circadian;*

Response: Urine was collected as clean-catch specimens at the time of clinical evaluation in order to maximize the detection of clinically-relevant KD markers. We have revised the manuscript to clarify this process (revised text in blue, pg. 14). We have also revised the manuscript to include measurements and normalization based on urine creatinine concentrations to account for potential variability in urine concentration that may be associated with physiologic factors such as circadian rhythms or disease-specific factors (revised Figs. 1A and B, revised Supp. Fig. 1). Creatinine-normalized meprin A and filamin C continue to show excellent diagnostic performance (ROC AUC 0.98, revised Fig. 1C).

Comment 3. *To validate the fidelity of the markers, it is recommended to analyze the urine peptide spectra of suspected KD patients before treatment such as corticosteroids and after the treatment to validate the correlation. Since the authors claim for identification of therapeutic targets of KD using their current protocol, the comparison of urine peptide spectra before and after treatment is essential;*

Response: Following the suggestion from Reviewer 1, we now exclusively focus on the diagnostic characteristics of meprin A and filamin C. Thus, attempting to identify any modified peptides upon corticosteroid treatment is beyond the scope of the revised version of the manuscript. Having said that, the point raised by Reviewer 2 is highly relevant and will be part of the follow-up studies where we will look more into the potential therapeutic target aspects of meprin A and filamin C.

Comment 4. *The authors should elaborate why elevated levels of filamin C and meprin in urine sample could be used as diagnostic markers for KD.*

Response: We have revised the manuscript to clarify the rationale and biologic basis for the use of meprin A and filamin C as diagnostic markers of KD (revised text in blue, pg. 11).

Referee #3

Comment 1. *The authors report the re-appearance of elevated levels of meprin A in the one patient that relapsed. What about levels of filamin C? It appears that the levels did not elevate (or were not tested for?). What, if any, implications do the authors think this has for the predictive role of filamin c?*

> Response: We agree with the referee that serial measurements of KD markers are very informative, revealing not only their diagnostic performance, but also possibly insights into disease pathophysiology. Unfortunately, we did not analyze filamin C levels in the serial specimens from this patient as we had only limited amounts of urine available at the time of analysis. We hope to do so in the future, as part of a planned study to prospectively collect serial specimens from patients with and without KD.

Comment 2. *What were the filamin C levels in the murine serum? The filamin C levels were elevated in human serum. Could this be related to the murine model, or a lowered predictive power of filamin c concentration in plasma (see above comment)? This may be worth some discussion?*

> Response: We have revised the manuscript to present these results, which show elevations of both meprin A and filamin C in the serum of disease mice (revised Fig. 4).

Comment 3. *Would the authors expect altered levels of IL-1 and IL-6 with an elevated level of meprin A in KD patients? Could this be tested for in the murine model?*

> Response: We agree with the referee completely. Indeed, IL-1 is highly elevated in both patients with KD and in the LCWE mouse model we used, as recently published by Lee *et al* (Interleukin-1β is crucial for the induction of coronary artery inflammation in a mouse model of Kawasaki disease. Circulation 125(12): 1542-50, 2012). Genetic experiments to determine the functional contribution of meprin A and interleukin signaling to the pathophysiology of Kawasaki disease are crucial and are important directions of future work.

2nd Editorial Decision 17 October 2012

Thank you for the submission of your revised manuscript to EMBO Molecular Medicine. We have now received the enclosed report from the referee who was asked to re-assess it in light of your previous submission.

As you will see, the reviewer is now supportive of publication and I am glad to let you know that we can proceed with the official acceptance of the manuscript after the following editorial points have been addressed:

- The description of all reported data that includes statistical testing must state the name of the statistical test used to generate error bars and P values, the number (n) of independent experiments underlying each data point (not replicate measures of one sample), and the actual P value for each test (not merely 'significant' or 'P < 0.05'). Please see http://onlinelibrary.wiley.com/journal/10.1002/(ISSN)1757-4684/homepage/ForAuthors.html#data2 for more information.

- For experiments involving human subjects the submission must include a statement that informed consent was obtained from all subjects and that the experiments conformed to the principles set out in the WMA Declaration of Helsinki [http://www.wma.net/en/30publications/10policies/b3/] and the NIH Belmont Report [http://ohsr.od.nih.gov/guidelines/belmont.html]. Please see our Guide to Authors for further information and provide the necessary information in the respective Material and Methods part.

Please submit your revised manuscript within two weeks. I look forward to seeing a revised form of

your manuscript as soon as possible.

Yours sincerely,

Editor
EMBO Molecular Medicine

***** Reviewer's comments *****

Referee #1 (Comments on Novelty/Model System):

The authors have adequately addressed both the urine protein quantification and urine creatinine normalization related concerns. Therefore, all my previous comments have been successfully addressed.
