# Supplementary material for: Urine proteomics for discovery of improved diagnostic markers of Kawasaki disease
Source: EMBO Mol Med. 2012 Dec 20;5(2):210–20. doi: 10.1002/emmm.201201494 (PMC3569638; doi:10.1002/emmm.201201494)

Supplementary Information for Kentsis et al. Urine proteomics for discovery of improved diagnostic markers of Kawasaki disease

Table of contents

Supp. Table 1: Proteins identified uniquely in the urine of patients with Kawasaki disease 2

Supp. Table 2: Processed proteomics data. 10

Supp. Table 3: Meprin A and Filamin C are significantly elevated in urine of patients with KD .. 11

Supp. Table 4: Meprin A and Filamin C are significantly elevated in serum of patients with KD 11

Supp. Fig. 1: Urine protein concentration (top) and creatinine concentration (bottom) in patients with and without KD. 12

Supp. Fig. 2: Urine meprin A levels in KD patients with and without pyuria. 13

Supp. Fig. 3: Urine filamin C levels in patients suspected of Kawasaki disease as a function of their C-reactive protein levels. 14

Supp. Fig. 4: Urine meprin A levels in patients suspected of Kawasaki disease as a function of their C-reactive protein levels. 15

Supp. Fig. 5: Urine filamin C levels in patients suspected of Kawasaki disease as a function of their erythrocyte sedimentation rates. 16

Supp. Table 1: Proteins identified uniquely in the urine of patients with Kawasaki disease

IPI ID	UniProt/SwissProt ID	HGNC symbol	Description
IPI00062003		ACAT1	acetyl-CoA acetyltransferase 1 [Source:HGNC Symbol;Acc:93]
IPI00414057		ACTA1	actin, alpha 1, skeletal muscle [Source:HGNC Symbol;Acc:129]
IPI00759776		ACTN1	actinin, alpha 1 [Source:HGNC Symbol;Acc:163]
IPI00019884	ACTN2_HUMAN	ACTN2	actinin, alpha 2 [Source:HGNC Symbol;Acc:164]
IPI00183703		AMICA1	adhesion molecule, interacts with CXADR antigen 1 [Source:HGNC Symbol;Acc:19084]
IPI00020019	ADIPO_HUMAN	ADIPOQ	adiponectin, C1Q and collagen domain containing [Source:HGNC Symbol;Acc:13633]
IPI00029733	AK1C1_HUMAN	AKR1C1	aldo-keto reductase family 1, member C1 (dihydrodiol dehydrogenase 1; 20-alpha (3-alpha)-hydroxysteroid dehydrogenase) [Source:HGNC Symbol;Acc:384]
IPI00293721	ARK73_HUMAN	AKR7A3	aldo-keto reductase family 7, member A3 (aflatoxin aldehyde reductase) [Source:HGNC Symbol;Acc:390]
IPI00298622	PPBI_HUMAN	ALPI	alkaline phosphatase, intestinal [Source:HGNC Symbol;Acc:437]
IPI00816309	ZA2G_HUMAN	AZGP1	alpha-2-glycoprotein 1, zinc-binding [Source:HGNC Symbol;Acc:910]
IPI00042580	APOO_HUMAN	APOO	apolipoprotein O [Source:HGNC Symbol;Acc:28727]
IPI00007068	ARP3B_HUMAN	ACTR3B	ARP3 actin-related protein 3 homolog B (yeast) [Source:HGNC Symbol;Acc:17256]
IPI00298306	ATM_HUMAN	ATM	ataxia telangiectasia mutated [Source:HGNC Symbol;Acc:795]
IPI00186972		AGBL5	ATP/GTP binding protein-like 5 [Source:HGNC Symbol;Acc:26147]
IPI00302644		ATP6V1C2	ATPase, H+ transporting, lysosomal 42kDa, V1 subunit C2 [Source:HGNC Symbol;Acc:18264]
IPI00003021	AT1A2_HUMAN	ATP1A2	ATPase, Na+/K+ transporting, alpha 2 polypeptide [Source:HGNC Symbol;Acc:800]
IPI00293460	ABCA1_HUMAN	ABCA1	ATP-binding cassette, sub-family A (ABC1), member 1 [Source:HGNC Symbol;Acc:29]
IPI00019906		BSG	basigin (Ok blood group) [Source:HGNC Symbol;Acc:1116]
IPI00744685		BTD	biotinidase [Source:HGNC Symbol;Acc:1122]
IPI00254408		BPTF	bromodomain PHD finger transcription factor [Source:HGNC Symbol;Acc:3581]
IPI00290089	CAD17_HUMAN	CDH17	cadherin 17, LI cadherin (liver-intestine) [Source:HGNC Symbol;Acc:1756]
IPI00020599	CALR_HUMAN	CALR	calreticulin [Source:HGNC Symbol;Acc:1455]
IPI00026185	CAPZB_HUMAN	CAPZB	capping protein (actin filament) muscle Z-line, beta [Source:HGNC Symbol;Acc:1491]
IPI00009823	CBPA1_HUMAN	CPA1	carboxypeptidase A1 (pancreatic) [Source:HGNC

			Symbol;Acc:2296]
IPI00009826	CBPB1_HUMAN	CPB1	carboxypeptidase B1 (tissue) [Source:HGNC Symbol;Acc:2299]
IPI00385428		CEACAM1	carcinoembryonic antigen-related cell adhesion molecule 1 (biliary glycoprotein) [Source:HGNC Symbol;Acc:1814]
IPI00027412	CEAM6_HUMAN	CEACAM6	carcinoembryonic antigen-related cell adhesion molecule 6 (non-specific cross reacting antigen) [Source:HGNC Symbol;Acc:1818]
IPI00658053	CATD_HUMAN	CTSD	cathepsin D [Source:HGNC Symbol;Acc:2529]
IPI00299150	CATS_HUMAN	CTSS	cathepsin S [Source:HGNC Symbol;Acc:2545]
IPI00385291		CD82	CD82 molecule [Source:HGNC Symbol;Acc:6210]
IPI00477763	MRCKB_HUMAN	CDC42BPB	CDC42 binding protein kinase beta (DMPK-like) [Source:HGNC Symbol;Acc:1738]
IPI00009619		CADM3	cell adhesion molecule 3 [Source:HGNC Symbol;Acc:17601]
IPI00413781		CXCL12	chemokine (C-X-C motif) ligand 12 [Source:HGNC Symbol;Acc:10672]
IPI00014625	CLCA1_HUMAN	CLCA1	chloride channel accessory 1 [Source:HGNC Symbol;Acc:2015]
IPI00298082	CLCA4_HUMAN	CLCA4	chloride channel accessory 4 [Source:HGNC Symbol;Acc:2018]
IPI00307485	CEL3B_HUMAN	CELA3B	chymotrypsin-like elastase family, member 3B [Source:HGNC Symbol;Acc:15945]
IPI00642792	CNDP2_HUMAN	CNDP2	CNDP dipeptidase 2 (metallopeptidase M20 family) [Source:HGNC Symbol;Acc:24437]
IPI00413344	COF2_HUMAN	CFL2	cofilin 2 (muscle) [Source:HGNC Symbol;Acc:1875]
IPI00743696	CO4A1_HUMAN	COL4A1	collagen, type IV, alpha 1 [Source:HGNC Symbol;Acc:2202]
IPI00021715	CO4A5_HUMAN	COL4A5	collagen, type IV, alpha 5 [Source:HGNC Symbol;Acc:2207]
IPI00329573	COCA1_HUMAN	COL12A1	collagen, type XII, alpha 1 [Source:HGNC Symbol;Acc:2188]
IPI00472110	CR1L_HUMAN	CR1L	complement component (3b/4b) receptor 1-like [Source:HGNC Symbol;Acc:2335]
IPI00332395	CPNE9_HUMAN	CPNE9	copine family member IX [Source:HGNC Symbol;Acc:24336]
IPI00002657	CPNE7_HUMAN	CPNE7	copine VII [Source:HGNC Symbol;Acc:2320]
IPI00377041	CSMD3_HUMAN	CSMD3	CUB and Sushi multiple domains 3 [Source:HGNC Symbol;Acc:19291]
IPI00249672	CUZD1_HUMAN	CUZD1	CUB and zona pellucida-like domains 1 [Source:HGNC Symbol;Acc:17937]
IPI00216569	CYTF_HUMAN	CST7	cystatin F (leukocystatin) [Source:HGNC Symbol;Acc:2479]
IPI00176698	CYC_HUMAN	CYCS	cytochrome c, somatic [Source:HGNC Symbol;Acc:19986]
IPI00071824	CKAP2_HUMAN	CKAP2	cytoskeleton associated protein 2 [Source:HGNC Symbol;Acc:1990]
IPI00376377	DHRS2_HUMAN	DHRS2	dehydrogenase/reductase (SDR family) member 2 [Source:HGNC Symbol;Acc:18349]
IPI00418512	DMBT1_HUMAN	DMBT1	deleted in malignant brain tumors 1 [Source:HGNC Symbol;Acc:2926]

IPI00028931	DSG2_HUMAN	DSG2	desmoglein 2 [Source:HGNC Symbol;Acc:3049]
IPI00007249	ENPP4_HUMAN	ENPP4	ectonucleotide pyrophosphatase/phosphodiesterase 4 (putative) [Source:HGNC Symbol;Acc:3359]
IPI00006690	PERE_HUMAN	EPX	eosinophil peroxidase [Source:HGNC Symbol;Acc:3423]
IPI00419721	EPMIP_HUMAN	EPM2AIP1	EPM2A (laforin) interacting protein 1 [Source:HGNC Symbol;Acc:19735]
IPI00376221	E41L5_HUMAN	EPB41L5	erythrocyte membrane protein band 4.1 like 5 [Source:HGNC Symbol;Acc:19819]
IPI00025491	IF4A1_HUMAN	EIF4A1	eukaryotic translation initiation factor 4A1 [Source:HGNC Symbol;Acc:3282]
IPI00010105	IF6_HUMAN	EIF6	eukaryotic translation initiation factor 6 [Source:HGNC Symbol;Acc:6159]
IPI00064917		FAM151A	family with sequence similarity 151, member A [Source:HGNC Symbol;Acc:25032]
IPI00554521	FRIH_HUMAN	FTH1	ferritin, heavy polypeptide 1 [Source:HGNC Symbol;Acc:3976]
IPI00339224		FN1	fibronectin 1 [Source:HGNC Symbol;Acc:3778]
IPI00289334	FLNB_HUMAN	FLNB	filamin B, beta [Source:HGNC Symbol;Acc:3755]
IPI00178352	FLNC_HUMAN	FLNC	filamin C, gamma [Source:HGNC Symbol;Acc:3756]
IPI00031708	FAAA_HUMAN	FAH	fumarylacetoacetate hydrolase (fumarylacetoacetase) [Source:HGNC Symbol;Acc:3579]
IPI00298383		FXYD2	FXYD domain containing ion transport regulator 2 [Source:HGNC Symbol;Acc:4026]
IPI00019383	GALK1_HUMAN	GALK1	galactokinase 1 [Source:HGNC Symbol;Acc:4118]
IPI00441550		GLB1	galactosidase, beta 1 [Source:HGNC Symbol;Acc:4298]
IPI00098026		GGT1	gamma-glutamyltransferase 1 [Source:HGNC Symbol;Acc:4250]
IPI00018169	IF_HUMAN	GIF	gastric intrinsic factor (vitamin B synthesis) [Source:HGNC Symbol;Acc:4268]
IPI00513929		GDI2	GDP dissociation inhibitor 2 [Source:HGNC Symbol;Acc:4227]
IPI00003929	GSTA3_HUMAN	GSTA3	glutathione S-transferase alpha 3 [Source:HGNC Symbol;Acc:4628]
IPI00639805		GSTM2	glutathione S-transferase mu 2 (muscle) [Source:HGNC Symbol;Acc:4634]
IPI00795622		GAPDH	glyceraldehyde-3-phosphate dehydrogenase [Source:HGNC Symbol;Acc:4141]
IPI00470823	GP2_HUMAN	GP2	glycoprotein 2 (zymogen granule membrane) [Source:HGNC Symbol;Acc:4441]
IPI00640867		GNAS	GNAS complex locus [Source:HGNC Symbol;Acc:4392]
IPI00306543	GDF15_HUMAN	GDF15	growth differentiation factor 15 [Source:HGNC Symbol;Acc:30142]
IPI00249267	H2AZ_HUMAN	H2AFZ	H2A histone family, member Z [Source:HGNC Symbol;Acc:4741]
IPI00419884	H3C_HUMAN	H3F3C	H3 histone, family 3C [Source:HGNC Symbol;Acc:33164]
IPI00009931	HDHD3_HUMAN	HDHD3	haloacid dehalogenase-like hydrolase domain containing 3 [Source:HGNC Symbol;Acc:28171]

IPI00657660		HBD	hemoglobin, delta [Source:HGNC Symbol;Acc:4829]
IPI00641229	IGHA2_HUMAN	IGHA2	immunoglobulin heavy constant alpha 2 (A2m marker) [Source:HGNC Symbol;Acc:5479]
IPI00553092		IGLV7-46	immunoglobulin lambda variable 7-46 (gene/pseudogene) [Source:HGNC Symbol;Acc:5930]
IPI00103356		ITGB2	integrin, beta 2 (complement component 3 receptor 3 and 4 subunit) [Source:HGNC Symbol;Acc:6155]
IPI00103436	ITLN2_HUMAN	ITLN2	intelectin 2 [Source:HGNC Symbol;Acc:20599]
IPI00011692	INVO_HUMAN	IVL	involucrin [Source:HGNC Symbol;Acc:6187]
IPI00166646	JPH3_HUMAN	JPH3	junctophilin 3 [Source:HGNC Symbol;Acc:14203]
IPI00001639	IMB1_HUMAN	KPNB1	karyopherin (importin) beta 1 [Source:HGNC Symbol;Acc:6400]
IPI00216136		KHK	ketohexokinase (fructokinase) [Source:HGNC Symbol;Acc:6315]
IPI00448751		KIAA1598	KIAA1598 [Source:HGNC Symbol;Acc:29319]
IPI00604711	KIF1A_HUMAN	KIF1A	kinesin family member 1A [Source:HGNC Symbol;Acc:888]
IPI00554498	LDHC_HUMAN	LDHC	lactate dehydrogenase C [Source:HGNC Symbol;Acc:6544]
IPI00016670	LTOR1_HUMAN	LAMTOR1	late endosomal/lysosomal adaptor, MAPK and MTOR activator 1 [Source:HGNC Symbol;Acc:26068]
IPI00009750	LEG4_HUMAN	LGALS4	lectin, galactoside-binding, soluble, 4 [Source:HGNC Symbol;Acc:6565]
IPI00788236	LAIR1_HUMAN	LAIR1	leukocyte-associated immunoglobulin-like receptor 1 [Source:HGNC Symbol;Acc:6477]
IPI00017940	LMBD2_HUMAN	LMBRD2	LMBR1 domain containing 2 [Source:HGNC Symbol;Acc:25287]
IPI00472013	1A31_HUMAN	HLA-A	major histocompatibility complex, class I, A [Source:HGNC Symbol;Acc:4931]
IPI00743503	1A26_HUMAN	HLA-A	major histocompatibility complex, class I, A [Source:HGNC Symbol;Acc:4931]
IPI00472057	1B73_HUMAN	HLA-B	major histocompatibility complex, class I, B [Source:HGNC Symbol;Acc:4932]
IPI00015988	HLAG_HUMAN	HLA-G	major histocompatibility complex, class I, G [Source:HGNC Symbol;Acc:4964]
IPI00013400	MMP7_HUMAN	MMP7	matrix metallopeptidase 7 (matrilysin, uterine) [Source:HGNC Symbol;Acc:7174]
IPI00167941	MDN1_HUMAN	MDN1	MDN1, midasin homolog (yeast) [Source:HGNC Symbol;Acc:18302]
IPI00004372	MEP1A_HUMAN	MEP1A	meprin A, alpha (PABA peptide hydrolase) [Source:HGNC Symbol;Acc:7015]
IPI00178015	MEP1B_HUMAN	MEP1B	meprin A, beta [Source:HGNC Symbol;Acc:7020]
IPI00103065	MITD1_HUMAN	MITD1	MIT, microtubule interacting and transport, domain containing 1 [Source:HGNC Symbol;Acc:25207]
IPI00418221	M3K6_HUMAN	MAP3K6	mitogen-activated protein kinase kinase kinase 6 [Source:HGNC Symbol;Acc:6858]
IPI00027201	MUC2_HUMAN	MUC2	mucin 2, oligomeric mucus/gel-forming [Source:HGNC Symbol;Acc:7512]

IPI00028553		MINPP1	multiple inositol-polyphosphate phosphatase 1 [Source:HGNC Symbol;Acc:7102]
IPI00019190	MYOC_HUMAN	MYOC	myocilin, trabecular meshwork inducible glucocorticoid response [Source:HGNC Symbol;Acc:7610]
IPI00217493	MYG_HUMAN	MB	myoglobin [Source:HGNC Symbol;Acc:6915]
IPI00844172		MYO6	myosin VI [Source:HGNC Symbol;Acc:7605]
IPI00023152	NALDL_HUMAN	NAALADL1	N-acetylated alpha-linked acidic dipeptidase-like 1 [Source:HGNC Symbol;Acc:23536]
IPI00220059	NDUB4_HUMAN	NDUFB4	NADH dehydrogenase (ubiquinone) 1 beta subcomplex, 4, 15kDa [Source:HGNC Symbol;Acc:7699]
IPI00009253	SNAA_HUMAN	NAPA	N-ethylmaleimide-sensitive factor attachment protein, alpha [Source:HGNC Symbol;Acc:7641]
IPI00514877		NCSTN	nicastrin [Source:HGNC Symbol;Acc:17091]
IPI00337541	NNTM_HUMAN	NNT	nicotinamide nucleotide transhydrogenase [Source:HGNC Symbol;Acc:7863]
IPI00451429		NIF3L1	NIF3 NGG1 interacting factor 3-like 1 (S. pombe) [Source:HGNC Symbol;Acc:13390]
IPI00017304	NOS2_HUMAN	NOS2	nitric oxide synthase 2, inducible [Source:HGNC Symbol;Acc:7873]
IPI00290416	OLA1_HUMAN	OLA1	Obg-like ATPase 1 [Source:HGNC Symbol;Acc:28833]
IPI00742748	OBSCN_HUMAN	OBSCN	obscurin, cytoskeletal calmodulin and titin-interacting RhoGEF [Source:HGNC Symbol;Acc:15719]
IPI00022324	O2AG1_HUMAN	OR2AG1	olfactory receptor, family 2, subfamily AG, member 1 [Source:HGNC Symbol;Acc:15142]
IPI00027720	LIPP_HUMAN	PNLIP	pancreatic lipase [Source:HGNC Symbol;Acc:9155]
IPI00016387	PCF11_HUMAN	PCF11	PCF11, cleavage and polyadenylation factor subunit, homolog (S. cerevisiae) [Source:HGNC Symbol;Acc:30097]
IPI00641498	PDZ1P_HUMAN	PDZK1	PDZ domain containing 1 [Source:HGNC Symbol;Acc:8821]
IPI00641244		PRDX1	peroxiredoxin 1 [Source:HGNC Symbol;Acc:9352]
IPI00219568	PGK2_HUMAN	PGK2	phosphoglycerate kinase 2 [Source:HGNC Symbol;Acc:8898]
IPI00021792	PA21B_HUMAN	PLA2G1B	phospholipase A2, group IB (pancreas) [Source:HGNC Symbol;Acc:9030]
IPI00026962	PA2GA_HUMAN	PLA2G2A	phospholipase A2, group IIA (platelets, synovial fluid) [Source:HGNC Symbol;Acc:9031]
IPI00432412			phospholipase inhibitor precursor [Source:RefSeq peptide;Acc:NP_001078943]
IPI00789401	PLSI_HUMAN	PLS1	plastin 1 [Source:HGNC Symbol;Acc:9090]
IPI00007248	PKHA6_HUMAN	PLEKHA6	pleckstrin homology domain containing, family A member 6 [Source:HGNC Symbol;Acc:17053]
IPI00107555		PFN2	profilin 2 [Source:HGNC Symbol;Acc:8882]
IPI00022213	PEPC_HUMAN	PGC	progastricsin (pepsinogen C) [Source:HGNC Symbol;Acc:8890]
IPI00792533		PGC	progastricsin (pepsinogen C) [Source:HGNC Symbol;Acc:8890]
IPI00514208		PTGDS	prostaglandin D2 synthase 21kDa (brain)

			[Source:HGNC Symbol;Acc:9592]
IPI00299571		PDIA6	protein disulfide isomerase family A, member 6 [Source:HGNC Symbol;Acc:30168]
IPI00010466	KPCB_HUMAN	PRKCB	protein kinase C, beta [Source:HGNC Symbol;Acc:9395]
IPI00296337	PRKDC_HUMAN	PRKDC	protein kinase, DNA-activated, catalytic polypeptide [Source:HGNC Symbol;Acc:9413]
IPI00332271	PTPRS_HUMAN	PTPRS	protein tyrosine phosphatase, receptor type, S [Source:HGNC Symbol;Acc:9681]
IPI00290350		PCDH19	protocadherin 19 [Source:HGNC Symbol;Acc:14270]
IPI00169326	PPTC7_HUMAN	PPTC7	PTC7 protein phosphatase homolog (S. cerevisiae) [Source:HGNC Symbol;Acc:30695]
IPI00293327	P2RX4_HUMAN	P2RX4	purinergic receptor P2X, ligand-gated ion channel, 4 [Source:HGNC Symbol;Acc:8535]
IPI00442204	MGAL1_HUMAN		Putative maltase-glucoamylase-like protein FLJ16351 [Source:UniProtKB/Swiss-Prot;Acc:Q6ZN80]
IPI00024282	RAB8B_HUMAN	RAB8B	RAB8B, member RAS oncogene family [Source:HGNC Symbol;Acc:30273]
IPI00166044	RPTOR_HUMAN	RPTOR	regulatory associated protein of MTOR, complex 1 [Source:HGNC Symbol;Acc:30287]
IPI00012622	RHG20_HUMAN	ARHGAP20	Rho GTPase activating protein 20 [Source:HGNC Symbol;Acc:18357]
IPI00152023	RNS11_HUMAN	RNASE11	ribonuclease, RNase A family, 11 (non-active) [Source:HGNC Symbol;Acc:19269]
IPI00219153	RL22_HUMAN	RPL22	ribosomal protein L22 [Source:HGNC Symbol;Acc:10315]
IPI00030179	RL7_HUMAN	RPL7	ribosomal protein L7 [Source:HGNC Symbol;Acc:10363]
IPI00745789	RSSA_HUMAN	RPSA	ribosomal protein SA [Source:HGNC Symbol;Acc:6502]
IPI00171771	RFWD2_HUMAN	RFWD2	ring finger and WD repeat domain 2 [Source:HGNC Symbol;Acc:17440]
IPI00433279	SLFN5_HUMAN	SLFN5	schlafen family member 5 [Source:HGNC Symbol;Acc:28286]
IPI00002606	ADSV_HUMAN	SCIN	scinderin [Source:HGNC Symbol;Acc:21695]
IPI00030385	SBP1_HUMAN	SELENBP1	selenium binding protein 1 [Source:HGNC Symbol;Acc:10719]
IPI00033583	SPB12_HUMAN	SERPINB12	serpin peptidase inhibitor, clade B (ovalbumin), member 12 [Source:HGNC Symbol;Acc:14220]
IPI00307466		SERPINB3	serpin peptidase inhibitor, clade B (ovalbumin), member 3 [Source:HGNC Symbol;Acc:10569]
IPI00413451	SPB6_HUMAN	SERPINB6	serpin peptidase inhibitor, clade B (ovalbumin), member 6 [Source:HGNC Symbol;Acc:8950]
IPI00394753	NRAM2_HUMAN	SLC11A2	solute carrier family 11 (proton-coupled divalent metal ion transporters), member 2 [Source:HGNC Symbol;Acc:10908]
IPI00008616	S12A7_HUMAN	SLC12A7	solute carrier family 12 (potassium/chloride transporters), member 7 [Source:HGNC Symbol;Acc:10915]
IPI00011981	S13A2_HUMAN	SLC13A2	solute carrier family 13 (sodium-dependent dicarboxylate transporter), member 2 [Source:HGNC Symbol;Acc:10917]

IPI00020542	S22AB_HUMAN	SLC22A11	solute carrier family 22 (organic anion/urate transporter), member 11 [Source:HGNC Symbol;Acc:18120]
IPI00171334	S22A4_HUMAN	SLC22A4	solute carrier family 22 (organic cation/ergothioneine transporter), member 4 [Source:HGNC Symbol;Acc:10968]
IPI00029268	SLC31_HUMAN	SLC3A1	solute carrier family 3 (cystine, dibasic and neutral amino acid transporters, activator of cystine, dibasic and neutral amino acid transport), member 1 [Source:HGNC Symbol;Acc:11025]
IPI00024248	SC5A5_HUMAN	SLC5A5	solute carrier family 5 (sodium iodide symporter), member 5 [Source:HGNC Symbol;Acc:11040]
IPI00760881	SC5AC_HUMAN	SLC5A12	solute carrier family 5 (sodium/glucose cotransporter), member 12 [Source:HGNC Symbol;Acc:28750]
IPI00003527	NHRF1_HUMAN	SLC9A3R1	solute carrier family 9 (sodium/hydrogen exchanger), member 3 regulator 1 [Source:HGNC Symbol;Acc:11075]
IPI00217882	SORT_HUMAN	SORT1	sortilin 1 [Source:HGNC Symbol;Acc:11186]
IPI00657938		SNX18	sorting nexin 18 [Source:HGNC Symbol;Acc:19245]
IPI00178767	ASM3A_HUMAN	SMPDL3A	sphingomyelin phosphodiesterase, acid-like 3A [Source:HGNC Symbol;Acc:17389]
IPI00291643	SPRY4_HUMAN	SPRYD4	SPRY domain containing 4 [Source:HGNC Symbol;Acc:27468]
IPI00000001	STAU1_HUMAN	STAU1	staufen, RNA binding protein, homolog 1 (Drosophila) [Source:HGNC Symbol;Acc:11370]
IPI00719690	SAM9L_HUMAN	SAMD9L	sterile alpha motif domain containing 9-like [Source:HGNC Symbol;Acc:1349]
IPI00514755		SDF4	stromal cell derived factor 4 [Source:HGNC Symbol;Acc:24188]
IPI00032826	ST134_HUMAN	ST13	suppression of tumorigenicity 13 (colon carcinoma) (Hsp70 interacting protein) [Source:HGNC Symbol;Acc:11343]
IPI00335277	SYPL1_HUMAN	SYPL1	synaptophysin-like 1 [Source:HGNC Symbol;Acc:11507]
IPI00289876	STX7_HUMAN	STX7	syntaxin 7 [Source:HGNC Symbol;Acc:11442]
IPI00642310		STXBP2	syntaxin binding protein 2 [Source:HGNC Symbol;Acc:11445]
IPI00298994	TLN1_HUMAN	TLN1	talin 1 [Source:HGNC Symbol;Acc:11845]
IPI00183938	TTC27_HUMAN	TTC27	tetratricopeptide repeat domain 27 [Source:HGNC Symbol;Acc:25986]
IPI00290452	TMBI1_HUMAN	TMBIM1	transmembrane BAX inhibitor motif containing 1 [Source:HGNC Symbol;Acc:23410]
IPI00023788	ENTK_HUMAN	TMPRSS15	transmembrane protease, serine 15 [Source:HGNC Symbol;Acc:9490]
IPI00010252	TRI33_HUMAN	TRIM33	tripartite motif containing 33 [Source:HGNC Symbol;Acc:16290]
IPI00018511	TBB8B_HUMAN		Tubulin beta-8 chain B [Source:UniProtKB/Swiss-Prot;Acc:A6NNZ2]
IPI00179709	TBA3C_HUMAN	TUBA3C	tubulin, alpha 3c [Source:HGNC Symbol;Acc:12408]
IPI00179709	TBA3C_HUMAN	TUBA3D	tubulin, alpha 3d [Source:HGNC Symbol;Acc:24071]

IPI00640721	1433B_HUMAN	YWHAB	tyrosine 3-monooxygenase/tryptophan 5-monooxygenase activation protein, beta polypeptide [Source:HGNC Symbol;Acc:12849]
IPI00011245	UBP29_HUMAN	USP29	ubiquitin specific peptidase 29 [Source:HGNC Symbol;Acc:18563]
IPI00783859		VPS13D	vacuolar protein sorting 13 homolog D (S. cerevisiae) [Source:HGNC Symbol;Acc:23595]
IPI00031655	VPS25_HUMAN	VPS25	vacuolar protein sorting 25 homolog (S. cerevisiae) [Source:HGNC Symbol;Acc:28122]
IPI00423568	RASK_HUMAN	KRAS	v-Ki-ras2 Kirsten rat sarcoma viral oncogene homolog [Source:HGNC Symbol;Acc:6407]
IPI00642108		WDR45	WD repeat domain 45 [Source:HGNC Symbol;Acc:28912]
IPI00550192	XPP3_HUMAN	XPNPEP3	X-prolyl aminopeptidase (aminopeptidase P) 3, putative [Source:HGNC Symbol;Acc:28052]
IPI00014513	TYY1_HUMAN	YY1	YY1 transcription factor [Source:HGNC Symbol;Acc:12856]
IPI00743220	ZN561_HUMAN	ZNF561	zinc finger protein 561 [Source:HGNC Symbol;Acc:28684]

Supp. Table 2: Processed proteomics data.

Raw data are available at the Peptide Atlas Repository (http://www.peptideatlas.org/PASS/PASS00104)

Processed data are supplied as separate file "emmm_201201494_sm_supptable_2.xlsx"

Supp. Table 3: Meprin A and Filamin C are significantly elevated in urine of patients with KD

Urine Marker	Non-Kawasaki disease	Kawasaki disease
**Filamin C (ng/ml)**	3.7 ± 3.0 [a, b]	19.2 ± 12.1 [a, b]
**Filamin C (ng/ml) / creatinine (mg/ml)**	0.056 ± 0.065 [a, b]	2.2 ± 11 [a, b]
**Meprin A (ng/ml)**	5.6 ± 5.7 [a, b]	50.2 ± 24.4 [a, b]
**Meprin A (ng/ml) / creatinine (mg/ml)**	0.10 ± 0.15 [a, b]	1.5 ± 1.2 [a, b]

Values in table represent mean ± standard deviation of untransformed measurements; statistical comparisons were carried out using analyses of log-transformed measurements. [a] $p < 0.05$ versus Kawasaki disease group, [b] $p < 0.05$ versus Kawasaki disease group.

Supp. Table 4: Meprin A and Filamin C are significantly elevated in serum of patients with KD

Serum Marker	Non-Kawasaki disease	Kawasaki disease
**Filamin C (ng/ml)**	6.6 ± 5.8 [a, b]	216.6 ± 707.8
**Meprin A (ng/ml)**	14.8 ± 9.1 [a, b]	1362.8 ± 3587.1

Values in table represent mean ± standard deviation of untransformed measurements; statistical comparisons were carried out using analyses of log-transformed measurements. [a] $p < 0.05$ versus Kawasaki disease group, [b] $p < 0.05$ versus Kawasaki disease group.

Supp. Fig. 1: Urine protein concentration (top) and creatinine concentration (bottom) in patients with and without KD. Urine protein concentration were measured using clinical urinalysis (0, 1, 2, 3).

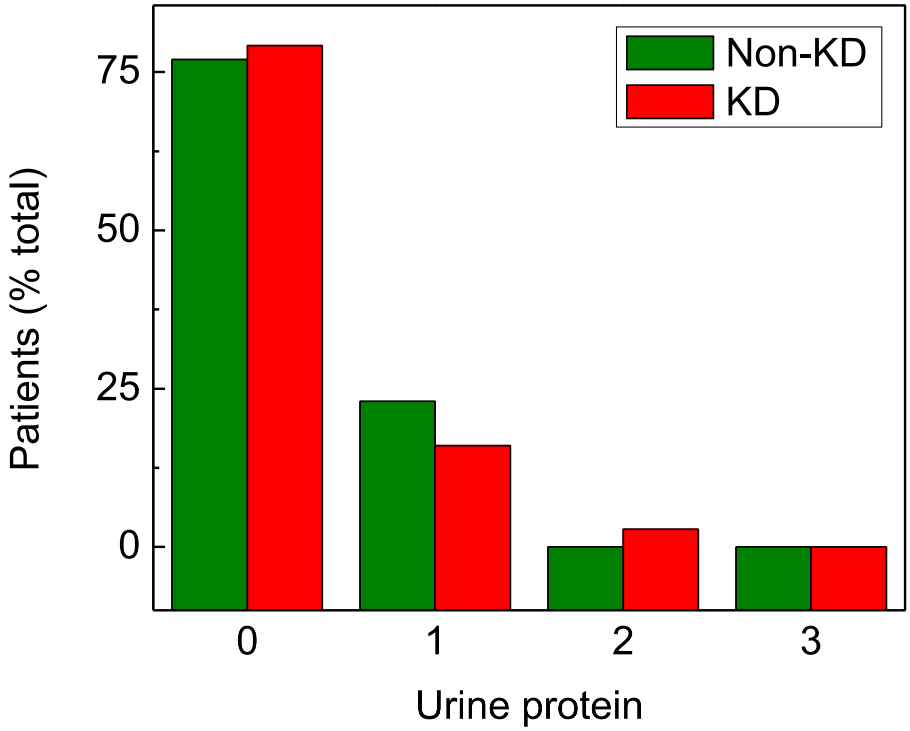

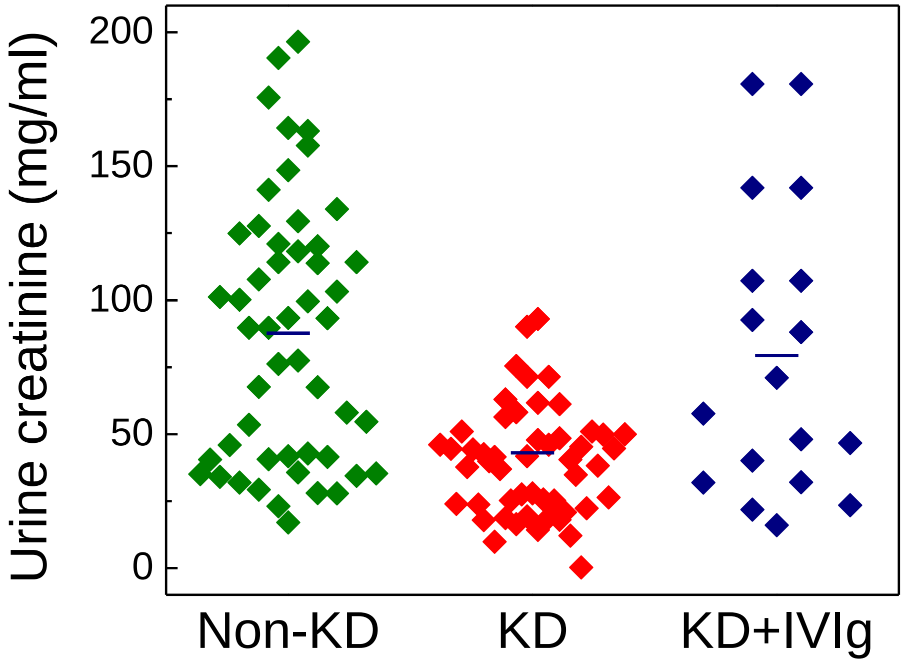

Supp. Fig. 2: Urine meprin A levels in KD patients with and without pyuria. Patients with Kawasaki disease who had <10 white blood cells per high-power field (WBC/hpf, no pyuria) have similar urine meprin A concentrations as those with >10 WBC/hpf (pyuria). $p > 0.15$.

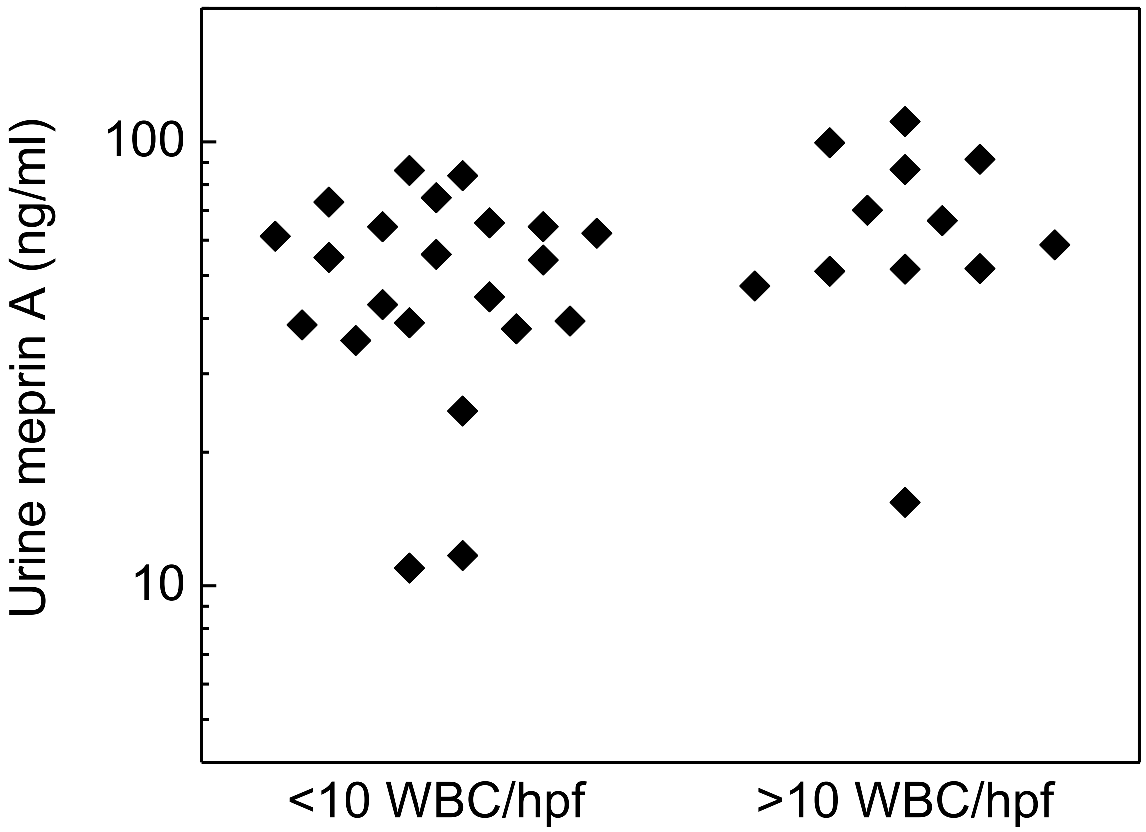

Supp. Fig. 3: Urine filamin C levels in patients suspected of Kawasaki disease as a function of their C-reactive protein levels.
Urine filamin C levels do not correlate with blood C-reactive protein levels. Adjusted $r^2 < 0.03$.

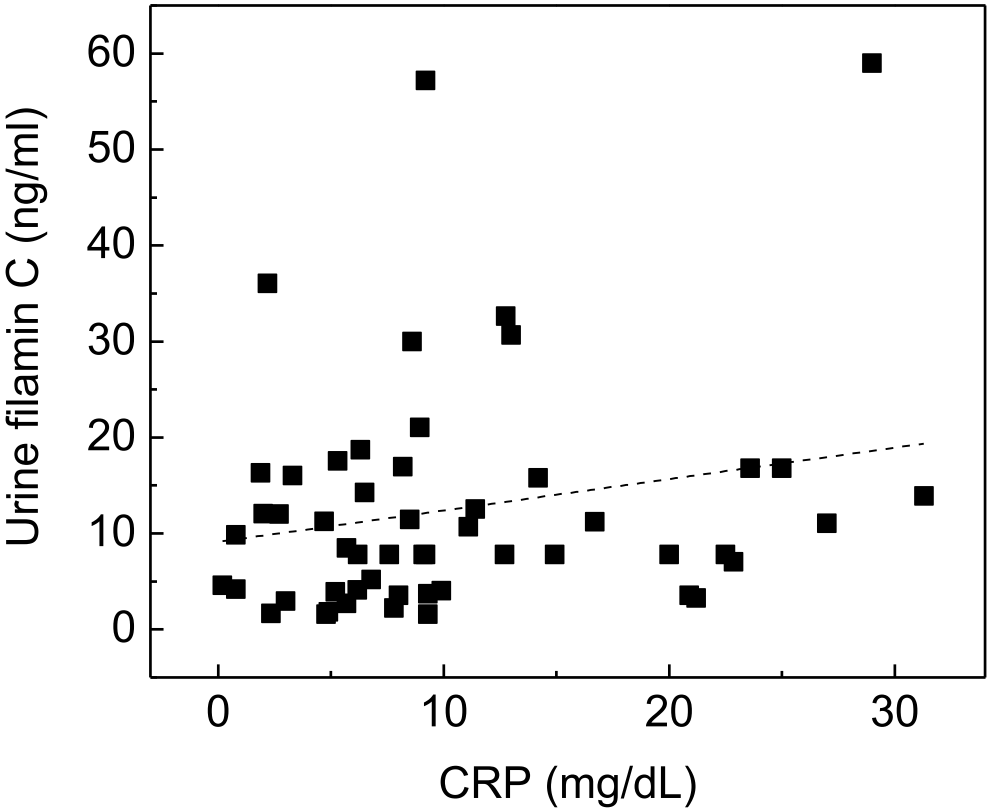

Supp. Fig. 4: Urine meprin A levels in patients suspected of Kawasaki disease as a function of their C-reactive protein levels.
Urine meprin A levels do not correlate with blood C-reactive protein levels. Adjusted $r^2 < 0.03$.

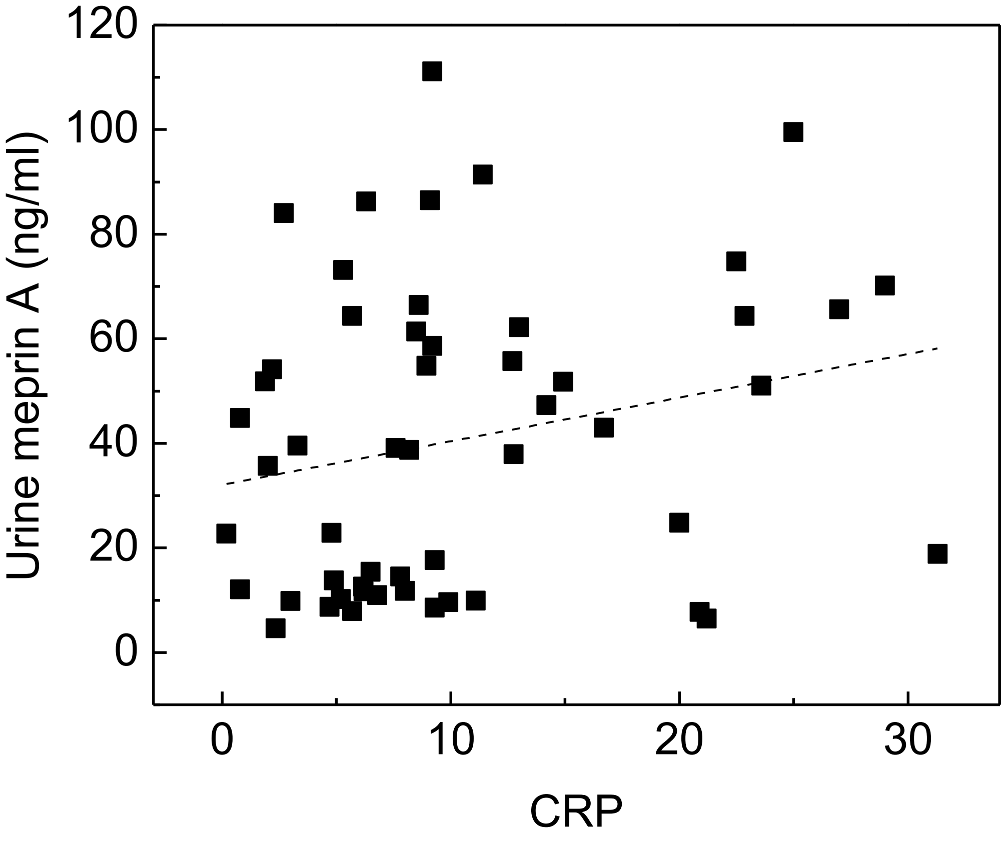

Supp. Fig. 5: Urine filamin C levels in patients suspected of Kawasaki disease as a function of their erythrocyte sedimentation rates. Urine filamin C levels do not correlate with ESR levels. Adjusted $r^2 < 0.01$.

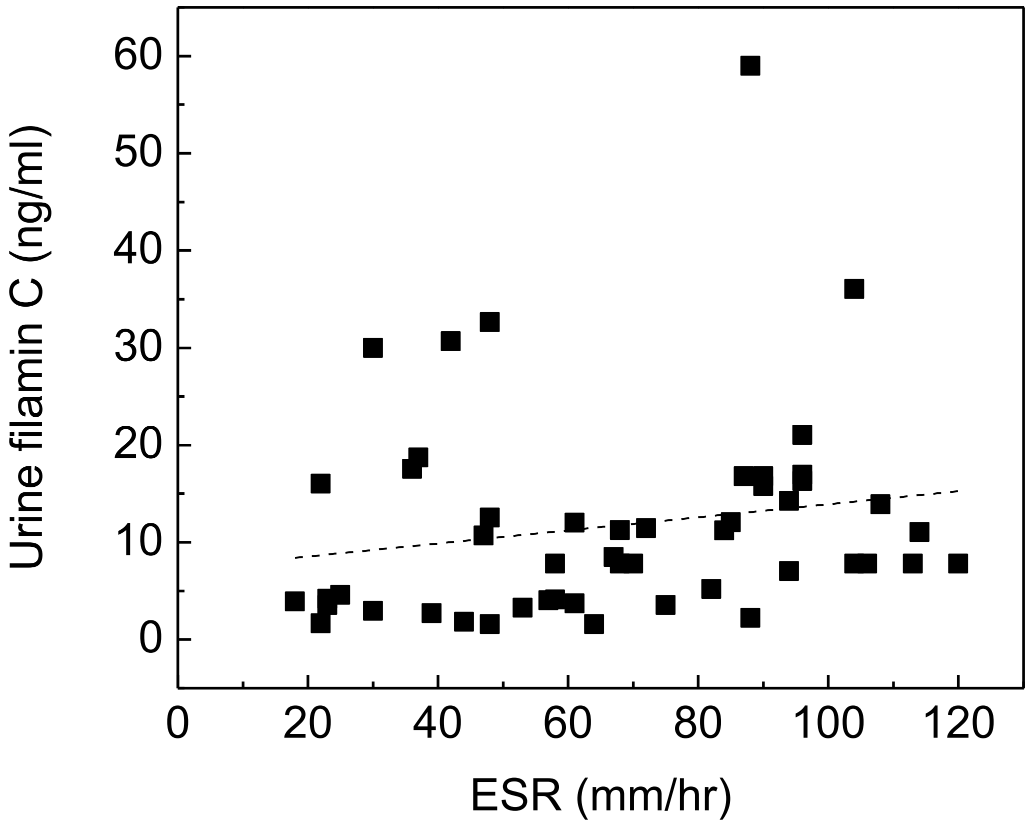

Supp. Fig. 6: Urine meprin A levels in patients suspected of Kawasaki disease as a function of their erythrocyte sedimentation rates. Urine meprin A levels do not correlate with ESR levels. Adjusted $r^2 < 0.05$.

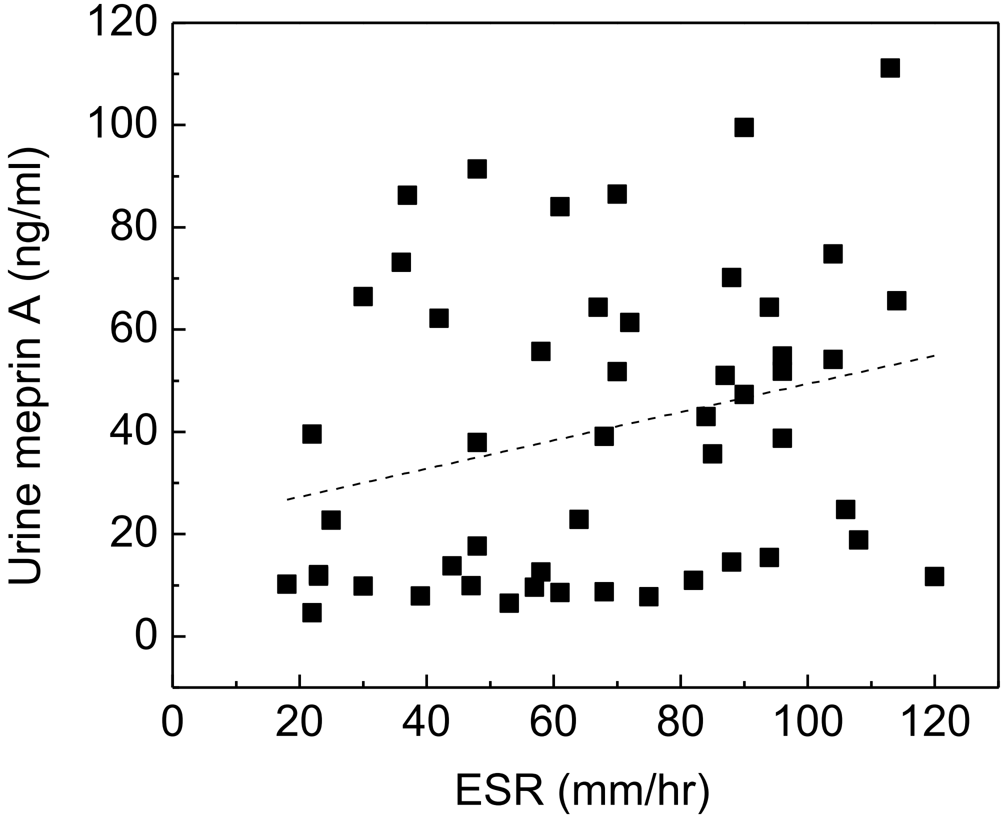

Supplement: Supplementary file 2 [file emmm0005-0210-SD2.pdf]
